# Epigenetic landscape influences the liver cancer genome architecture

**DOI:** 10.1038/s41467-018-03999-y

**Published:** 2018-04-24

**Authors:** Natsuko Hama, Yasushi Totoki, Fumihito Miura, Kenji Tatsuno, Mihoko Saito-Adachi, Hiromi Nakamura, Yasuhito Arai, Fumie Hosoda, Tomoko Urushidate, Shoko Ohashi, Wakako Mukai, Nobuyoshi Hiraoka, Hiroyuki Aburatani, Takashi Ito, Tatsuhiro Shibata

**Affiliations:** 1 Division of Cancer Genomics, National Cancer Center Research Institute, Chuo-ku, Tokyo, 104-0045 Japan; 2 Department of Biochemistry, Kyushu University Graduate School of Medical Sciences, Higashi-ku, Fukuoka, 812-8582 Japan; 3 Genome Science Division, Research Center for Advanced Science and Technology, The University of Tokyo, Tokyo, 153-0041 Japan; 4 Laboratory of Molecular Medicine, Human Genome Center, The Institute of Medical Science, The University of Tokyo, Minato-ku, Tokyo, 108-8639 Japan; 5 Division of Pathology and Clinical Laboratories, National Cancer Center Hospital, Chuo-ku, Tokyo, 104-0045 Japan

## Abstract

The accumulations of different types of genetic alterations such as nucleotide substitutions, structural rearrangements and viral genome integrations and epigenetic alterations contribute to carcinogenesis. Here, we report correlation between the occurrence of epigenetic features and genetic aberrations by whole-genome bisulfite, whole-genome shotgun, long-read, and virus capture sequencing of 373 liver cancers. Somatic substitutions and rearrangement breakpoints are enriched in tumor-specific hypo-methylated regions with inactive chromatin marks and actively transcribed highly methylated regions in the cancer genome. Individual mutation signatures depend on chromatin status, especially, signatures with a higher transcriptional strand bias occur within active chromatic areas. Hepatitis B virus (HBV) integration sites are frequently detected within inactive chromatin regions in cancer cells, as a consequence of negative selection for integrations in active chromatin regions. Ultra-high structural instability and preserved unmethylation of integrated HBV genomes are observed. We conclude that both precancerous and somatic epigenetic features contribute to the cancer genome architecture.

## Abstract (web summary)

Genomic aberrations contribute to the development of cancer; however, their interdependence remains poorly understood. Here the authors analyze liver cancer samples to find correlation between epigenetic features and genetic aberrations including somatic substitutions, mutation signatures, and HBV integration sites.

## Introduction

Whole cancer genome sequencing identified novel types of cancer drivers, including mutations and structural alterations in gene regulatory elements such as the promoter, untranslated region, and enhancer^1–3^, underscoring the importance of understanding epigenetic contexts simultaneously. Genetic and epigenetic alterations interdependently contribute to oncogenesis; however, how the processes underlying cancer-driving genetic events such as somatic mutation, structural rearrangement, and onco-virus genome integration are associated with or affected by the epigenetic features of the cancer genome remains poorly understood.

Hepatocellular carcinoma (HCC) is the six for cancer incidence and third as a cause of cancer-related death^4^. Approximately 300,000 new cases annually are associated with hepatitis B or C virus (HBV and HCV) infection^5^. HBV, a DNA virus that integrates its genome into the hepatocyte genome of infected humans, is the most prevalent and highest risk factor for HCC^5^. Virus genome insertions and step-wise somatic accumulation of genetic and epigenetic alterations during clonal evolution promote hepatocarcinogenesis^6^.

Here, whole-genome and methylome sequencing were used to elucidate the effect of the epigenetic context on the definition of the liver cancer genome architecture, including individual mutational signatures and clonal selection of viral genome integrations. These results suggest that precancerous and somatic epigenetic features play important roles in making the cancer genome, and understanding the underlying mechanisms would facilitate the identification of epigenetic driver events and carcinogenic processes involved in hepatocarcinogenesis.

## Results

### Chromatin context-dependent hypo-methylation in HCC

To simultaneously evaluate somatic genetic changes, viral integration events, and DNA methylation patterns in HCC genomes, we selected samples from HBV-positive patients, including three paired samples of tumors and their corresponding non-cancerous liver tissues, as well as two additional tumor samples (Supplementary Tables 1 and 2). We then performed whole-genome bisulfite sequencing (WGBS) of all eight samples and whole-genome sequencing (WGS) in both tumors (50.2 × average sequence coverage) and the corresponding lymphocyte genomes (49.8 × average sequence coverage) by short-read Illumina sequencing (all five pairs of cases), along with long-read WGS of one tumor (15.6 × sequence coverage) and lymphocyte (11.4 × sequence coverage) pair using the PacBio RS sequencer. The methylation data obtained by WGBS was significantly (*r* = 0.92–0.95, *P* < 2.2e–16, Pearson’s correlation test) concordant with that determined by array-based analysis (Supplementary Fig. 1a). To delineate the chromatin contexts, we utilized 15 epigenomic segments showing different histone configurations among the segments in normal adult liver or HCC cells (HepG2), as reported in the ENCODE project database^7^. These segments are hereafter referred to as E1–E15 for normal liver and C1–C15 for cancer cells (Supplementary Fig. 2). One genome yield 491,144 regions of E1–E15 or 561,497 regions of C1–C15.

Analysis of the bisulfite sequencing data revealed that an average of 71% of the covered cytosines at CpG sites were methylated in the non-cancerous liver genome, and the methylation level (56% on average) was significantly decreased (*P* = 0.016, Welch’s *t*-test) in the tumor genome. The decrease in methylation level increased in proportion to the distance from the CpG islands (Supplementary Figs. 1, 3, and 4).

**Fig. 1:**
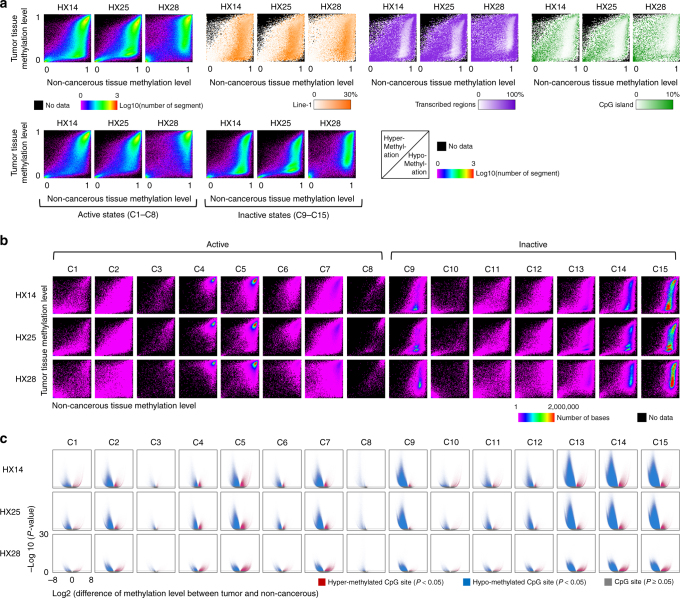
Hypo- and hyper-methylation of three paired hepatocellular carcinoma genomes. **a** In the upper panel, the left three plots show the methylation levels of the regions of tumor genomes (*y*-axis) or non-cancerous genomes (*x*-axis) delimited according to the 15 epigenomic segments. The color indicates the number of region. The orange plots show the density of LINE-1, purple plots show the density of transcribed regions, and green plots show the density of CpG islands. These densities were calculated for each fraction of the left three images, which were divided into 100 × 100 areas. In the lower panels, the genomic regions of the upper left three plots are shown separately for active and inactive chromatin state. **b** Methylation levels of tumor and non-cancerous genomes in the 15 epigenomic segments. The color indicates the number of bases. **c** Volcano plot of the differences in the methylation levels of CpG sites between tumor and non-cancerous genomes. Each dot represents a CpG site. Red indicates that the methylation level in the tumor genome is significantly (*P* < 0.05 using Fisher’s exact test) higher than in the non-cancerous genome, blue indicates lower, and gray indicates *P* ≥ 0.05. The *x*-axis indicates the log2 fold-change of the methylation level; the y-axis indicates the minus log10 of the *P* value

Analysis of the methylation data according to the 15 epigenomic segments of HCC cells identified large (up to 19.2 Mb) hypo-methylated regions that were specific to HCC cells (Supplementary Fig. 5a). These regions were enriched in repeated sequences, especially long interspersed element-1 (LINE-1) sequences, and poor in transcribed genomic regions and CpG islands (Fig. 1a and Supplementary Fig. 5b). The methylation level in the cancer genome was decreased in characteristic epigenomic segments, including in active chromatic genome regions that were annotated with proximal promoters (C2), a transcribed state at the 5′ and 3′ ends of genes showing both promoter and enhancer signatures (C3), enhancers (C7), and a state associated with zinc finger protein genes (C8), and in inactive regions annotated with heterochromatin (C9), repressed Polycomb (C13 and C14) and quiescent/low (C15) (Fig. 1b, c, Supplementary Table 3).

### Methylation level-dependent mutational density in HCC

Human cells have tissue-specific epigenomic marks that contribute to distinct gene expression and the biological function of each tissue^7^. In addition, the number of mutations and the spectrum of mutational signatures vary among cancers of different tissue origins^8^. These findings suggest the possibility that the epigenetic features of each tissue affect the mutational process during tumorigenesis.

**Fig. 2:**
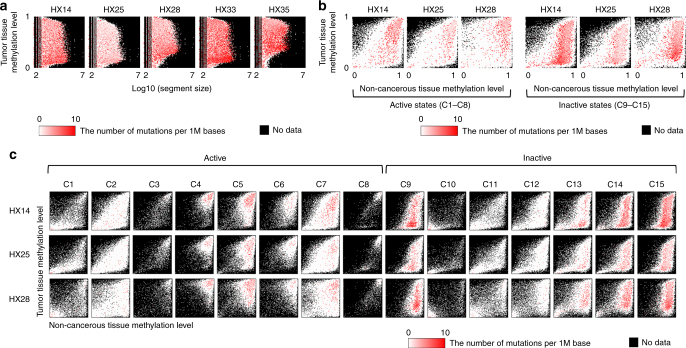
Density of mutations in the different epigenetic states in hepatocellular carcinoma. **a** The distribution difference of somatic mutations that depending on the differences in size (*x*-axis) and tumor methylation level (*y*-axis) of the region delimited by 15 epigenomic segments. Red indicates the number of mutations per million bases calculated for each fraction of the images in Supplementary Fig. 5a, which were divided into 100 × 100 areas. **b** Comparison of the distribution of somatic mutations between the active or inactive chromatin status. The *x*-axis indicates the methylation level of the non-cancerous liver genome and the *y*-axis indicates that of the tumor. The accumulation of mutations was higher in hypo-methylated regions in the inactive chromatin. Red indicates the number of mutations per million bases calculated for each fraction of the images of the lower panel of Fig. 1a, which were divided into 100 × 100 areas. **c** Density of mutations in the 15 epigenomic segments. Red indicates the number of mutations per million bases calculated for each fraction of the images of Fig. 1b, which were divided into 100 × 100 areas

To determine whether methylation status is associated with the distribution of somatic mutations, we examined the correlation between somatic mutation density and methylation level. In all paired samples, a significantly higher somatic mutation density (*P* < 2.2e–16, *Χ*^2^-test) was observed in the genomic areas showing different methylation levels between non-cancerous and cancerous liver samples. These areas were highly methylated in non-cancerous liver samples, whereas methylation was low in cancer samples (Fig. 2a, b). Further analysis of the distribution of mutation density in the 15 epigenomic segments identified two specific types of genomic regions harboring higher somatic mutation densities: (1) transcribed and enhancer genomic regions (C4–7) with active histone marks that were highly methylated in both non-cancerous liver and tumor samples (open chromatin with high methylation); and (2) heterochromatic and Polycomb-repressed areas (C9, C13–15) with inactive histone marks that were highly methylated in non-cancerous liver samples and showed low methylation in tumor samples (closed chromatin with somatic hypo-methylation) (Fig. 2b, c). These data suggest that somatic mutations may occur preferentially in highly methylated regions of the non-cancerous liver genome. These findings also indicate that the chromatin status may regulate the frequency of somatic mutations in liver cancer genomes.

### Epigenetic context-dependent mutational processes in HCC

**Fig. 3:**
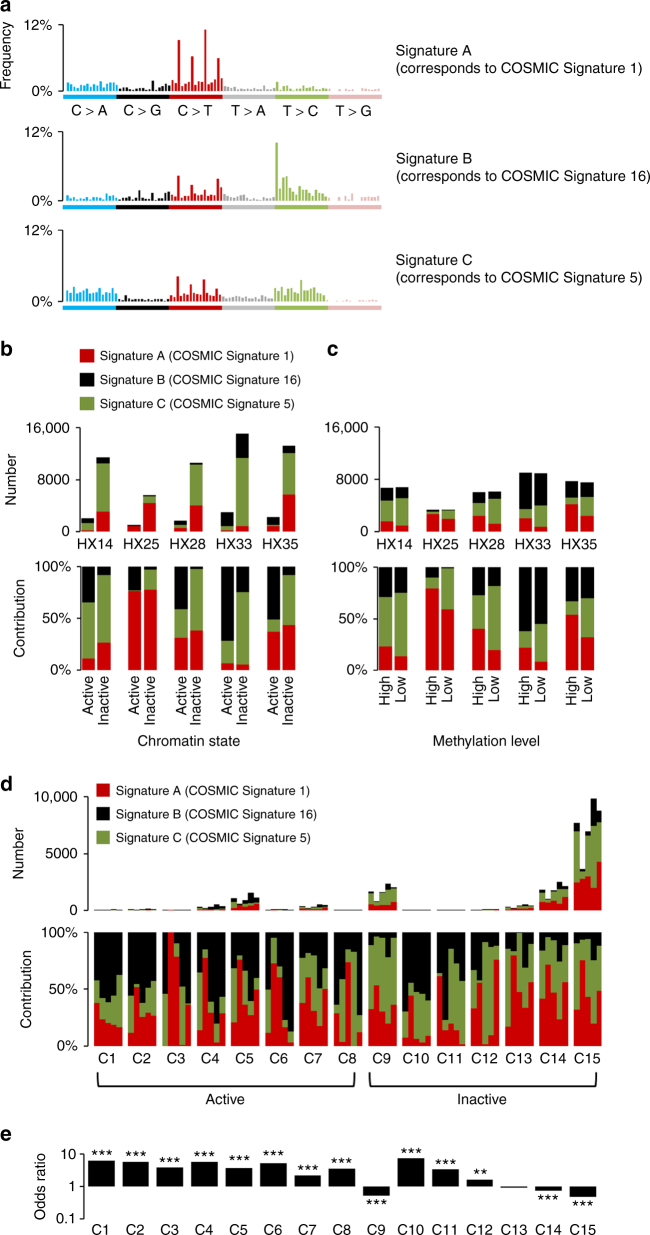
Mutation signature analysis of five hepatocellular carcinoma samples in different chromatin states or with different methylation levels. **a** Frequencies of the 96 substitution patterns of three extracted mutational signatures. The stability of the signature and the most similar COSMIC signature are shown in Supplementary Table 4. **b** Contribution of the three mutational signatures to each tumor sample according to the chromatin state. The *y*-axis indicates the number or contributions of each signature in the mutations. In each sample, significant differences were observed between active and inactive chromatin states (*P* < 2.2e–16, Pearson’s *Χ*^2^-test). The contribution of Signature B (which corresponds to COSMIC Signature 16) in active state was significantly higher than that in the inactive state (*P* < 2.2e–16, Pearson’s *Χ*^2^-test). **c** The contribution of three mutational signatures in genomic regions of different methylation levels. In each sample, the contribution was significantly different between high and low methylation levels (*P* < 2.2e–16, Pearson’s *Χ*^2^-test). The proportions of Signature A (which corresponds to COSMIC Signature 1) and B in highly methylated regions was significantly higher than the proportions in low methylated regions (*P* < 2.2e–16 in Sig. A, *P* < 2.8e–05 in Sig. B, Pearson’s *Χ*^2^-test). **d** The contributions of three signatures in 15 epigenomic segments. **e** Odds ratio of Signature B in a particular segment compared with the other segments. *P < 0.05, **P < 0.005, ***P < 0.0005 tested with Fisher’s t-test

Multiple somatic mutational processes that are induced by endogenous and exogenous factors occur in HCC;^8,9^ however, the role of the epigenetic context in these processes remains unclear. We first compared somatic substitution patterns among epigenetic segments. Both C > A/G > T and C > T/G > A substitutions occurred significantly more frequently in inactive chromatin areas, whereas T > G/A > C and T > C/A > G substitutions were more frequent in active chromatin areas (*P* < 0.05, Fisher’s exact test, Supplementary Fig. 6). To determine whether epigenetically uneven mutational processes contribute to the distribution of somatic substitutions, we compared mutational signatures^8^ among 15 epigenomic segments. Three mutational signatures with high stability were extracted from five HCC genomes (Methods); of these, two corresponded to COSMIC Signatures 1 (Signature A) and COSMIC Signature 16 (Signature B) in the COSMIC database (Fig. 3a and Supplementary Table 4)^8^. Analysis of the correlation with methylation status showed that COSMIC Signature 1, characterized by a high prevalence of C > T mutations at CpG sites and caused by the deamination of 5-methyl-cytosine, occurred preferentially in highly methylated areas of the genome. By contrast, COSMIC Signature 16, which is characterized by a high prevalence of T > C mutations and is unique to HCC^8^, occurred significantly more frequently in highly methylated and active chromatin areas in all cases (Fig. 3b, c). Furthermore, among the 15 epigenomic segments, COSMIC Signature 16 was enriched in genomic regions containing transcribed regions (such as C3, C4, and C5) and transcription start sites (C1, C2, C10, and C11), as well as enhancers (C6 and C7) (Fig. 3d, e). Analysis of the transcribed genomic areas revealed a markedly unbalanced distribution of mutations between the transcribed and un-transcribed strands in COSMIC Signature 16, indicating the presence of transcription-coupled repair, as previously reported^8^ (Supplementary Fig. 7a). In addition to this strand-specific effect of COSMIC Signature 16, the two signatures (COSMIC Signatures 1 and 16) reproducibly showed a chromatin context-dependent distribution (Supplementary Fig. 7b-e).

**Fig. 4:**
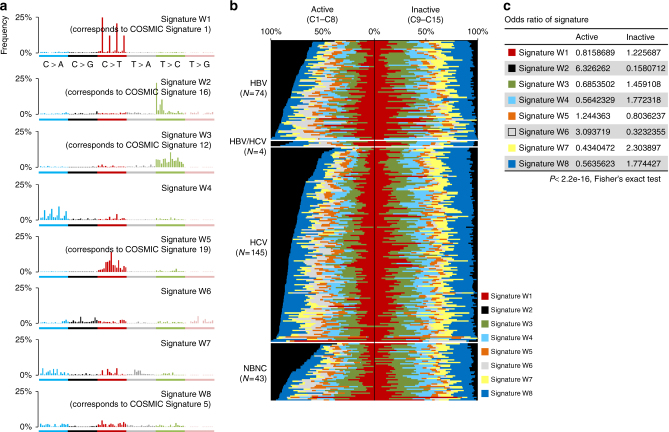
Mutational signature analysis of 266 hepatocellular carcinomas. **a** Eight mutational signatures identified from active or inactive chromatin states of 266 HCCs. The *y*-axis indicates the frequency of each of the 96 substitution patterns. The stability of the signature and the most similar COSMIC signature are shown in Supplementary Table 4. **b** Contribution of the eight mutational signatures to each tumor in active and inactive chromatin states. The *y*-axis indicates the number or the contribution of mutations in each signature. HBV: hepatitis B virus-related HCCs, HCV: hepatitis C virus-related HCCs, NBNC: HCCs without HBV or HCV infection. The differences in the odds ratios among viral features are shown in Supplementary Table 5. **c** Odds ratio of the contribution of the signature in active and inactive chromatin

We further attempted to verify these correlations in a larger cohort of WGS data from 266 HCC samples with diverse etiological backgrounds^9^. Eight mutational signatures (W1–8) were extracted from this data set (Fig. 4a, Supplementary Table 4, Supplementary Figs. 8 and 9), and the contribution of each signature was compared between the active and inactive chromatin status. A significant enrichment of W2 (corresponding to Signature B above and COSMIC Signature 16) in active chromatin areas (odds ratio: 6.32) was reproducibly observed irrespective of hepatitis virus status (Fig. 4b, c). Signatures W6 (odds ratio: 3.09) and W5 (which corresponds to COSMIC Signature 19) (odds ratio: 1.24) were enriched in the active histone-marked genome (Fig. 4b, c), whereas signatures W7 (odds ratio: 2.30), W4, W1 (which corresponds to COSMIC Signature 1), W3 (which corresponds to COSMIC Signature 12) and W8 occurred more frequently in inactive chromatin segments (Fig. 4b, c). The unique enrichment of COSMIC Signature 16 in specific epigenomic segments observed in five cases was also confirmed in this larger data set (Supplementary Fig. 9). In Fig. 4b, the contribution of COSMIC Signature 16 (signature W2) in the active chromatin state was higher and that of signatures W1, 6 and 7 was lower in non-hepatitis B and -C (NBNC) than that of viral positive groups (Supplementary Table 5). A similar trend for COSMIC Signature 16 was observed when mutational signatures were extracted with strand features (Supplementary Table 4, Supplementary Figs. 10, 11 and 12). Importantly, the intensities of the transcribed strand biases in these mutational signatures demonstrated a significant positive correlation with their tendency to occur in the active histone-marked genome (Supplementary Fig. 10d, *P* = 0.00207, Pearson’s correlation test). These findings suggest that mutational processes occur non-randomly, and that the intensity of strand biases is significantly associated with their preferential presence in the active chromatin genome in HCC.

### HBV genome integration and epigenetic signature

**Fig. 5:**
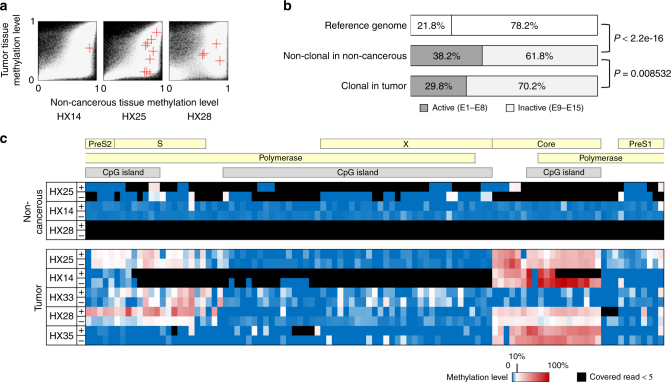
Integration and methylation of HBV genomes in hepatocellular carcinoma genomes. **a** Comparison of the methylation levels of the regions of tumor genomes (*y*-axis) that included the HBV-integrated sites with the methylation levels of the same region of non-cancerous genomes (*x*-axis). A red cross indicates the position of a region including the integration of HBV. In the background, a white dot shows a genomic region, and a black indicates no data. **b** Proportion of the HBV-integrated sites in active or inactive chromatin areas. The integrated sites were detected using whole-exome and oncovirome sequencing. Reference genome: the proportion of the base numbers of chromatin states in the normal adult liver defined in ENCODE;^7^ non-clonal: the proportion of the numbers of integration sites (tag count ≥2, ≤3) in 108 non-cancerous liver samples; clonal: the number of integration sites (tag count ≥5) in 102 tumor samples. The *P *value was calculated using Fisher’s test. **c** The methylation levels of all CpG sites in the HBV genome detected by whole-genome bisulfite sequencing of HCC samples

The HBV genome is a circular double-stranded DNA of ~ 3200 bases in length consisting of protein-coding regions, promoters, enhancers, and CpG islands^10,11^. The HBV genome integration process is associated with host DNA nicking and recombination^12,13^. However, the DNA methylation of integrated HBV sequences and the HCC genome around HBV integration sites remain to be thoroughly explored. We hypothesized that the HBV integration process could also be associated with chromatin status and may be more frequent in active chromatic genomes. To assess this, we first used paired-end reads from WGS data and identified HBV genome integration sites in five tumors. The integration sites were preferentially located in tumor-specific hypo-methylated regions with inactive histone marks in normal hepatocytes (Fig. 5a, Supplementary Table 6). This result suggested two hypotheses for HBV genome integration sites. First, the HBV genome may preferentially integrate into inactive epigenetic areas of the normal hepatocyte genome. Alternatively, HBV genome integration into active epigenetic areas could be negatively selected for during hepatocarcinogenesis. To test these hypotheses, non-clonal HBV genome integration events in non-cancerous hepatocytes were isolated from HBV-infected patients. Hybrid pair-reads mapping to both virus and human genomes were enriched using virus genome capture followed by deep sequencing (Methods). A total of 108 non-cancerous liver tissues and 102 HCC samples from the same patients^14^ were analyzed, and 1010 non-clonal integration events in non-cancerous tissues and 476 clonal integration events in HCCs were identified. Non-clonal HBV integrations in non-cancerous liver tissues were significantly (*P* < 2.2e–16, Fisher’s exact test) enriched in the transcriptionally active chromatin regions, whereas the clonal integration sites in tumors were located significantly more frequently in the inactive chromatin regions (Fig. 5b, *P* = 0.0085, Fisher’s exact test). This suggests that although substantial HBV integration occurs in open and transcribing epigenetic regions, these integration events are negatively selected for in cancerous tissues, probably because of their deleterious effects on hepatocyte growth. In line with this hypothesis, genes close to the non-clonal HBV integration sites frequently encoded metabolic enzymes and transporters that are highly expressed and play important physiological roles in hepatocytes (Supplementary Tables 7 and 8). This suggests that disruption of these genes would create a growth disadvantage.

The methylation status of the integrated HBV genome in HCC was also examined using WGBS data. An average of >96.9 × (25.8–230.9×) sequence coverage of HBV genomes was achieved by WGBS in tumor samples. WGBS revealed that the integrated HBV genome was broadly unmethylated (the CpG methylation level was 9.7% on average), with a slight tumor-specific increase in methylation (22.1% on average) in two CpG island regions (Fig. 5c).

### Somatic structural alterations and epigenetic context

**Fig. 6:**
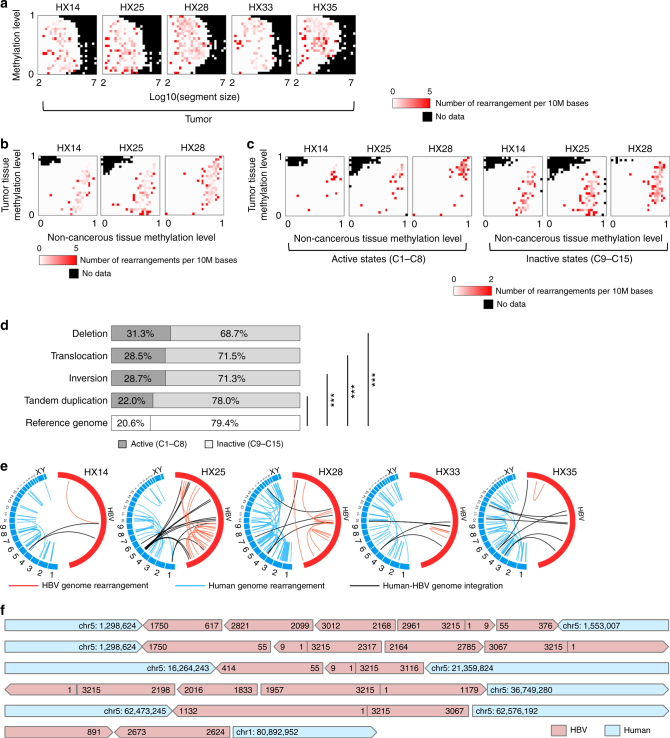
Somatic structural alterations of the cancer and integrated virus genomes. **a** The density of somatic rearrangements in five tumor samples. The color indicates the number of rearrangements per 10 million bases calculated for each fraction of the images in Supplementary Fig. 5a, which were divided into 25 × 25 areas. The *x*-axis indicates the size of the region and the* y*-axis indicates the methylation level of the region. All rearrangements in these samples are shown in Supplementary Data 1. **b** Accumulation of rearrangements according to different methylation levels of tumor (*y*-axis) and non-cancerous (*x*-axis) genomes. Red indicates the number of rearrangements per 10 million bases calculated for each fraction of the images in the upper left in Fig. 1a. **c** Comparison of the accumulation of rearrangements between active and inactive chromatin areas. **d** The proportion of somatic rearrangements in active and inactive chromatin areas analyzed using whole-genome sequencing (WGS) of 21 HCCs. In the active areas, deletions, translocations, and inversions were significantly enriched compared with those in the inactive areas. *P < 0.05, **P < 0.005, ***P < 0.0005 using the *Χ*^2^-test. **e** Structural rearrangements of the HBV and human genomes in five samples. Red line: HBV internal rearrangement; black: HBV and human genome breakpoint; blue: human genome rearrangement. **f** Six long reads from PacBio WGS of the HX25 tumor, including the complex rearrangement of viral and human genome sequences. The integrated or rearranged site was validated by at least two short reads obtained by the Illumina platform

To determine whether structural alterations were also associated with the epigenetic context, we compared the WGS and WGBS data of five cases. The density of somatic rearrangement breakpoints did not differ significantly between hyper- and hypo-methylated areas of the tumor genome (Fig. 6a, b). However, considering the somatic changes in methylation and epigenetic marks, the breakpoints of somatic rearrangements were enriched in the two aforementioned genomic areas: active chromatin areas that were highly methylated in both tumor and non-cancerous liver samples and tumor-specific hypo-methylated areas of the genome with inactive chromatin marks (Fig. 6b, c), where the density of somatic mutations was also high (Fig. 2b). Classification according to the type of rearrangement showed that somatic inversions, translocations and deletions occurred significantly more frequently in active chromatin genomic areas (Fig. 6d and Supplementary Data 1). By contrast, somatic retro-transposed positions of LINE-1 elements were not enriched in the active chromatin areas of the genome in two of three tumors (Supplementary Fig. 13), which suggested inter-tumor diversity. Collectively, these findings imply that the epigenetic context was diversely associated with structural alterations in HCC.

### Structural rearrangements of integrated HBV genomes

The positions of HBV genome integration were associated with somatic structural rearrangements (Supplementary Fig. 14). Previous studies report occasional alterations of integrated HBV genomes^15,16^. We therefore explored whether structural alterations of HBV genomes in the tumor genome may play a role in the host genome structural rearrangements. Paired reads that both mapped to the HBV genome were used to detect HBV genome rearrangements in all five HCC genomes (Fig. 6e), and the frequency of these rearrangements (per Mb) was extremely higher than that of the tumor genome (42,815-fold increase on average, *P* < 2.2e–16 for all five samples, using the *Χ*^2^-test) (Supplementary Table 9).

To verify the rearrangements in the HBV genomes, we obtained long-read (the average length of a sequenced read was 1783 kb) WGS data for one pair of HCC and non-cancerous liver genomes (HX25). Based on the 16 × coverage of PacBio long-read WGS (Methods), we *de novo* constructed six independent long consensus reads (5425–10,251 kb) of the integrated HBV genome, three of which covered both integrated ends, and three of which covered a single integrated end. These reads perfectly confirmed the presence of the rearrangements (deletions, inversions, and duplications) detected by the Illumina short reads (Fig. 6f).

To further confirm this finding, we performed HBV capture sequencing of a larger HCC cohort and identified more than five rearrangements in 74% (45/61) of cases. The presence of frequent (more than five) HBV rearrangements was not associated with the mutation status of known driver genes including TP53 (Supplementary Fig. 15). Characteristically, tumors with frequent HBV genome rearrangements harbored significantly fewer total somatic mutations (*P* = 0.04, Pearson’s correlation test), suggesting that HBV genome instability-associated genetic alterations may complement part of the driver events in HCC.

## Discussion

Comprehensive genome and methylome sequencing of liver cancer samples identified two epigenetically unique genomic areas in which somatic genetic aberrations (both mutation and rearrangement) were enriched. One region was tumor-specifically hypo-methylated and showed an inactive chromatic genome, including a late replicating region (e.g., heterochromatin). Previous studies in other cancer types also report a higher somatic mutation rate in the hypo-methylated cancer genome^17–19^. The other region was actively transcribed and contained a highly methylated gene body region that included potential therapeutic targets^20^. These include areas that are vulnerable to genetic assaults such as DNA damaging agents, are repair-deficient during replication, and in part positively selected during carcinogenesis. By contrast, mutation density was lower in the hypo-methylated and active chromatic region including oncogenic enhancers^21^, which upregulate aberrant transcription in various cancer types^2,22^. These results imply that, in addition to be their direct involvement in carcinogenesis, both precancerous and somatic epigenetic features have instructive roles in the establishment of the cancer genome architecture.

Further analysis revealed the underlying molecular mechanisms determining the uneven distribution of somatic mutations in the epigenetic context. COSMIC Signature 1, induced by the deamination of 5-methyl-cytosine, occurred more frequently in the highly methylated genome. By contrast, COSMIC Signature 16, which shows marked transcription-coupled repair, preferentially occurred in the highly methylated and active chromatin areas of the genome, including transcribed and enhancer regions. Our analysis also identified a significant correlation between the degree of transcription-coupled strand bias in mutational signatures and their preferential occurrence in the active chromatin areas, especially in the liver-characteristic COSMIC Signature 16. DNA adduct formation, which is physically recognized by the nucleotide excision repair complex^23,24^ and prone to mis-repair during the mutation generating process, is affected by chromatin status^25,26^. Our results suggest that genome-wide DNA accessibility regulated by histone modification could be a structural and/or physical limiting factor for the DNA modification-mediated mutational processes underlying the epigenetic context-dependent distribution of these signatures. The mutational signature induced by ultraviolet-mediated DNA cross-linking in melanoma is affected by transcription factor binding or DNase hypersensitivity^27^, and other signatures in breast cancer are affected by nucleosome occupancy^28^. Taken together, these results highlight the intensive influence of epigenetic features on mutational processes in human cancer.

The identification of the epigenetic contexts of non-clonal and clonal HBV integration sites indicated that the initial HBV integration may preferentially occur at active chromatin areas of the normal hepatocyte genome, probably because of the accessibility of DNA modifying proteins and viral DNA. However, during carcinogenesis, and most likely prior to clonal evolution, cells in which vital genes are disrupted by viral integration may be subject to negative selection; in support of this notion, the clonal integration sites in the tumor genome were more frequently located in constitutively methylated/closed chromatin areas. This illustrates the mechanism by which the epigenetic context and clonal evolution cooperatively and chronologically give rise to the cancer genome.

This study also provides the finest mapping of structural changes in integrated HBV genomes by long-read sequencing, revealing the ultra-high instability and preserved unmethylation of the HBV genome integrated in the HCC genome. Such local superior genome instability, which is negatively correlated to the total number of somatic mutations, may be associated with an increase in somatic structural rearrangements near HBV integration sites^14,29^.

In conclusion, this integrative analysis identified interdependency between genetic, viral, and epigenetic alterations in liver cancer. Understanding the underlying molecular mechanisms would facilitate the identification of epigenetic driver events as well as carcinogenic processes and ultimately contribute to genome-based treatment and prevention.

## Methods

### Clinical samples and DNA extraction

The clinical and pathological features of five patients who provided the HCC samples used for WGBS are shown in Supplementary Tables 1 and 2. HBV-related tumors were defined by the presence of hepatitis B surface antigen (HBsAg) in serum. All subjects had undergone partial hepatectomy, and the diagnosis of HCC was confirmed by pathologists based on samples containing >80% of viable tumor cells. High molecular weight genomic DNA was extracted from fresh-frozen tumor and non-cancerous specimens and blood as described previously^14^. All subjects who agreed to participate in the study provided informed consent according to the ICGC guidelines^30^ (http://www.icgc.org). Ethical committees at the National Cancer Center Japan approved this work (Project number: 2005-030).

### WGBS and methylation analysis

WGBS libraries were prepared using the post-bisulfite adaptor tagging^31^ protocol. First, 100 ng of genomic DNA was bisulfite-treated with an EZ DNA Methylation Gold kit from Zymo Research. To estimate the bisulfite conversion rate, 1 ng of unmethylated lambda DNA from Promega was mixed into each sample before the bisulfite conversion. Two rounds of random primer extension were performed to prepare libraries for single-end sequencing. The first round was performed on the bisulfite-treated DNA and the second round on the DNA synthesized in the first reaction. The concentration of the templates was determined by qPCR using Library Quantification Kits from KAPA Biosystems. Single-end sequencing with 100 cycle reactions was performed using an Illumina HiSeq 2000 instrument. All sequence data were mapped to the UCSC human genome 19 (hg19) using BMap (http://itolab.med.kyushu-u.ac.jp/BMap/index.html) as described previously^31^. The methylation level of each cytosine was calculated only when it was covered by at least five reads. Epigenomic segments of the genome were identified using the 15 epigenomic segments defined in the HepG2 hepatoblastoma cell line or human adult liver cells by the Roadmap Epigenomics Consortium^7^ (Supplementary Fig. 2) and plotted using the gplots package in R (https://cran.r-project.org/web/packages/gplots/index.html).

### WGS

Short-fragment libraries (insert size range of 300–500 bp) were prepared from 1.5–3 μg of genomic DNA from tumors and lymphocytes. Sequencing was performed on the Illumina Genome Analyzer II and HiSeq 2000 platforms with paired-end reads of 50–125 bp according to the manufacturer’s instructions^14^.

### Somatic point mutation and short indel calls

Paired-end reads were aligned to the human reference genome (GRCh37) using the Burrows-Wheeler Aligner (BWA)^32^. Probable PCR duplications, in which paired-end reads were aligned to the same genomic positions, were removed using SAMtools^33^ and a program developed in-house^14^. To identify somatic point mutations and short indels, the mapping result files were converted to the pileup format using SAMtools. The details of the analytical informatics methods used were reported in our previous studies^9,34,35^.

### Analysis of mutation patterns and signatures

The number of each of 96 possible somatic substitution types (C > A/G > T, C > G/G > C, C > T/G > A, T > A/A > T, T > C/A > G and T > G/A > C, for the bases immediately 5′ and 3′ to each substitution) or 192-substitution types considered the transcriptional strand in transcribing regions was counted for each sample. The frequency of each of these substitutions was determined by dividing each count by the total number of substitutions and was adjusted by the frequency of each tri-nucleotide pattern in the human reference genome. Non-negative matrix factorization (NMF) was applied to the 96- or 192-substitution pattern using published software^8^, running 1000 iterations of NMF with each NMF run iterated until convergence was achieved (10,000 iterations without change) or until the maximum number of 1,000,000 iterations was reached. Comparison with the COSMIC signature was performed by cosine similarity as described previously^36^.

### Detection of somatic structural alterations

Fifty bp paired-end reads were used for rearrangement analysis, because they contain longer spacers than 100 bp paired-end reads. Therefore, 100 bp paired-end reads were cut to generate 50 bp paired-end reads. To detect structural alterations, paired-end reads for which both ends aligned uniquely to the human reference genome, but with improper spacing, orientation, or both were considered as previously reported^35^.

### HBV integration sites and rearrangement breakpoints

Detection by WGS: To identify HBV integrations using the WGS data, the viral genome sequences for HBV (NC_003977.1), HPV-16 (NC_001526), HPV-18 (NC_001357), and HTLV-1 (NC_001436) were downloaded from NCBI and included as reference files when reads were mapped by bowtie^37^. The paired-end reads, that were perfectly matched to the human genome, were removed, and the rest were re-aligned to the reference files using the BLASTN^38^ program with the -F F -culling_limit of three options. To identify viral integration sites and the breakpoints of HBV genome rearrangements, only the paired-end reads that satisfied all of the following conditions were used: the read was mapped with a read length * 0.20, a 25 bp minimal alignment length and 95% alignment identity, allowing one gap; at least 80% of the read length was aligned to the human or HBV genome; the overlap or gap between two alignments in the read was set at a maximum of 10 bp. Then, the reads that included a portion of the HBV genomic sequence were classified into two groups: those for which the junction of two alignments was located in the sequenced region of paired-end reads, or those for which the junction was located between the paired-end reads. From the former group of reads, the genomic coordinates and read-orientation at each junction were extracted. If three or more reads shared nearly the same coordinates (allowing an ambiguity of 5 bp) and the same orientation, the junction was defined as the breakpoint of viral integration or HBV genome rearrangement. In the latter group, the paired-end reads were categorized by their orientation and insert size: forward–forward, reverse–reverse, reverse–forward, and forward–reverse for which the spacing was too large (> maximum insert size: 550 bp). The reads in each category were clustered according to their coordinates if the minimum distance between the edges of two reads was <50 bp (HBV genome) or 100 bp (human genome) in both of the paired-end reads. If the cluster size was over the maximum insert size, the cluster was discarded. If the cluster included at least four paired-end reads, we considered that to indicate a viral integration site or the breakpoint of an HBV rearrangement generated in a non-sequenced region between the paired-end reads.

Detection by whole-exome and oncovirome sequencing: The sequences of hepatitis B viral genomes from NCBI were included in the reference files when sequence reads were mapped by BWA. To achieve more precise HBV mapping, all reads were mapped to the HBV reference sequence using the q-gram and Smith-Waterman methods. HBV integration sites were clustered by genomic position as previously described^14^. HBV integration sites detected by two or three supporting reads were considered to be non-clonal integration in non-cancerous livers. Genes in which HBV integrated non-clonally in non-cancerous samples but did not contain integrations in tumors were identified for pathway analysis, and the Investigate Gene Sets tools on the Gene Set Enrichment Analysis^39,40^ website (http://software.broadinstitute.org/gsea/msigdb/annotate.jsp) was used with the C5, Bio Carta, KEGG, and Reactome gene sets. Sixty-one HBsAg-positive HCC samples were selected from the samples analyzed by HBV genome rearrangement as reported previously^35^.

### Long-read sequencing

Whole-genome long sequence data were obtained using the single-molecule real-time (SMRT) sequencing method with the PacBio RSII instrument. Long-insert sequence libraries (SMRTbell-10kb) were prepared from 20 μg of paired tumor and non-cancerous tissue DNA from HX25, enriched to >4 kb using BluePippin, loaded on SMRT Cell v3, and sequenced with P4C2 chemistry according to the manufacturer’s instructions.

The sequence reads (tumor: 15.64 average depth, 1783 bp average mapped read length, and 85.35% mapped accuracy; non-cancerous: 11.36 average depth, 4082 bp average mapped read length, and 84.99% mapped accuracy) were mapped using BLASR^41^. Long reads that included at least two HBV integration sites or included both an integration site and a breakpoint of structural rearrangement of HBV genome sequences were selected. Each integration site or breakpoint in the read was validated by the detection of integration or rearrangement of HBV using the WGS short reads described above.

### Detection of chromosomal transposition driven by Line-1

To identify chromosomal transposition driven by Line-1, we used 50–125 bp paired-end reads of WGS with an average insert size of 500–600 bp prepared from three tumors and paired lymphocytes (HX14, HX25, and HX28).

First, low quality reads were filtered out;

- Mapping quality is <30,
- Containing contiguous 10 or more bases whose sequence quality is <35,
- PCR duplicated reads.

Next, chromosomal transposition candidates were extracted from filtered data using two algorithms: one was based on soft-clip mapping and the other used irregular arrangements of paired-end reads. The first algorithm detected the breakpoint, which was located on sequences of paired-end reads. BWA-MEM with -T 0 option was used for mapping to the hg19(GRCh37) reference genome, and only reads soft-clipped on two different chromosomes were retained.

To select authentic alignment, the following four criteria were used: (i) if two or more alignment regions were detected in the reference genome, the mapping quality of the primary alignment must exceed at least 10% of that of others; (ii) the nucleotide base of the edge of the read must be aligned; (iii) the number of mismatches is less than 3% of the alignment length; and (iv) one-half or more of the read length must be aligned. Then, the genomic coordinates of soft-clipped junctions and the read-orientations of both sides of the reads were extracted from each soft-clip alignment. If a junction supported three or more reads (allowing an ambiguity of 5 bp with a gap or an overlap) and did not appear in control read data prepared from lymphocytes, it was outputted as the transposition breakpoint.

The second algorithm detected the breakpoint located between paired-end reads, and only both end reads mapped to different chromosomes were used. The detailed methods used to detect chromosomal transposition were described previously^35^. After the candidates were outputted from the two algorithms, they were merged in reference to their read IDs to avoid overlapping the reads.

Finally, the breakpoint candidates were annotated regardless of the presence of Line-1-driven transpositions. RepeatMasker (ver.4.0.6) was used for annotation of the Line-1 family sequence, and the criteria predefined in Reference 42 were used for assessment of driver transposable elements.

### Data availability

Sequencing data are available at the European Genome-phenome Archive (EGA) under accession EGAS00001002230 (WGBS), EGAS00001000671 (WGS) and EGAS00001000389 (whole-exome and oncovirome sequencing). Please contact the data access committee (DAC) of the International Cancer Genome Consortium (ICGC) for access to the data. All other remaining data are available within the Article and Supplementary Files, or available from the authors upon request.

## Electronic supplementary material

Supplementary Information

Description of Additional Supplementary Files

Supplementary Data 1
