# Supplementary material for: Epigenetic landscape influences the liver cancer genome architecture
Source: Nat Commun. 2018 Apr 24;9:1643. doi: 10.1038/s41467-018-03999-y (PMC5915380; doi:10.1038/s41467-018-03999-y)
Supplement: Supplementary file 1 — Supplementary Information [file 41467_2018_3999_MOESM1_ESM.pdf]

Supplementary Table 1:
Clinical and pathological information of the 5 hepatocellular carcinoma (HCC) analyzed

ID	Age	Gender	Virus infection	T[a]	N	M	Edmond-son grade	Tumor size (mm)	Portal vein invasion	Hepatic vein invasion	Hepatic artery invasion	Bile duct invasion	Liver fibrosis[b]	Alcohol intake[c]	Smok-ing	Prog-nosis[d]	Overall survival (month)
HX14	56	M	HBV	3	0	0	3	63	1	0	0	0	3	1	1	1	53
HX25	41	M	HBV	3	0	0	2	65	1	0	0	0	3	0	NA	1	24
HX28	58	M	HBV	4	0	0	2	52	1	0	0	0	3	3	0	0	55
HX33	63	M	HBV	3	0	0	2~3	50	1	0	0	0	1~2	1	1	1	29
HX35	68	M	HBV	4	0	0	2~3	50	1	1	0	0	1~2	2	0	0	19

a; TNM staging in UICC
b; Fibrosis in non-cancerous liver tissue is determined according the New Inuyama Classification.
c; 0: no alcohol intake, 1: social drinker, 2: ~60g every day, 3: 60g and more every day
d; 0: survial, 1: death from liver cancer, 2: surgery-related death, 3: death from other diseases

Supplementary Table 2:
Summary of whole-genome bisulfite sequencing of five hepatocellular carcinoma samples.

		Tumor					Non-cancerous		
		HX14	HX25	HX28	HX33	HX35	HX14	HX25	HX28
Depth of top		19.1	16.5	4.8	23.4	21.9	18.1	28.3	10.4
Depth of bottom		19.2	16.5	4.8	23.4	21.9	18.1	28.4	10.4
CpG	Depth	29.7	19.3	6.1	31.9	28.1	20.4	32.7	12.0
	Covered region* (%)	74.4	76.3	47.8	78.5	76.8	76.7	81.3	70.0
	Methylation level (%)	47.8	44.9	56.8	47.3	70.8	71.2	72.1	74.4
	Correlation with array**	0.932	0.931	0.916	0.943	0.946	No data	No data	No data
CHG***	Depth	26.5	19.8	6.0	30.2	27.4	21.2	33.7	12.3
	Covered region* (%)	77.2	80.5	49.0	82.4	80.7	81.1	85.8	74.0
	Methylation level (%)	1.0	1.1	1.3	1.1	1.0	1.3	1.2	1.0
CHH***	Depth	21.8	17.7	5.1	25.9	23.7	19.3	30.2	11.0
	Covered region* (%)	72.6	77.6	41.8	79.8	77.5	78.5	84.4	69.7
	Methylation level (%)	0.7	0.8	1.1	0.8	0.8	1.0	0.9	0.7

*Supported with at least five reads.
**Infinium HumanMethylation450K, P < 2.2e-16 with Pearson's correlation test.
***Cytosines in non-CpG context (where H = A, T or C).

Supplementary Table 3 | Hypo-methylated epigenomic segments in tumor tissue of three paired HCC samples

	HX14		HX25		HX28	
	Segment*	Hypo-methylation**	Segment*	Hypo-methylation**	Segment*	Hypo-methylation**
Active	C8	50.14	C8	56.35	C8	34.77
	C2	36.95	C2	38.24	C2	24.30
	C3	30.58	C7	36.98	C3	23.33
	C7	30.22	C3	31.09	C7	18.99
	C6	22.90	C6	24.24	C6	12.62
	C5	10.73	C5	21.84	C5	7.35
	C4	6.62	C4	17.90	C4	4.52
	C1	6.52	C1	5.82	C1	2.86
Inactive	C13	62.72	C9	68.52	C9	40.90
	C9	61.53	C13	66.90	C13	33.07
	C14	54.20	C14	62.17	C14	30.94
	C15	47.19	C15	56.79	C15	29.89
	C12	41.54	C12	48.62	C12	22.74
	C11	24.99	C11	24.68	C11	12.31
	C10	5.47	C10	3.55	C10	1.79

*The 15 epigenomic segments of HepG2 cell line defined in ENCODE project (see Supplementary Fig. 2 and Reference 7)

**The percentage of the number of significantly (p-value <0.05 with Fisher's exact test) hypo-methylated CpG site

Supplementary Table 4:
The stability and the most similar COSMIC signatures of the mutational signatures

Signature of five samples shown in **Figure 3a**	Stability	Most similar COSMIC signature[*]	Description of COSMIC signature[**]
Signature A	0.968806	Signature 1	Cancer types: Signature 1 has been found in all cancer types and in most cancer samples.<br>Proposed aetiology: Signature 1 is the result of an endogenous mutational process initiated by spontaneous deamination of 5-methylcytosine.<br>Additional mutational features: Signature 1 is associated with small numbers of small insertions and deletions in most tissue types.
Signature B	0.872883	Signature 16	Cancer types: Signature 16 has been found in liver cancer.<br>Proposed aetiology: The aetiology of Signature 16 remains unknown.<br>Additional mutational features: Signature 16 exhibits an extremely strong transcriptional strand bias for T>C mutations at ApTpN context, with T>C mutations occurring almost exclusively on the transcribed strand.
Signature C	0.826825	Signature 5	Cancer types: Signature 5 has been found in all cancer types and most cancer samples.<br>Proposed aetiology: The aetiology of Signature 5 is unknown.<br>Additional mutational features: Signature 5 exhibits transcriptional strand bias for T>C substitutions at ApTpN context.

Signature of 266 samples shown in **Figure 4a**	Stability	Most similar COSMIC signature[*]	Description of COSMIC signature[**]
Signature W1	0.999251	Signature 1	(See above)
Signature W2	0.99577	Signature 16	(See above)
Signature W3	0.992675	Signature 12	Cancer types: Signature 12 has been found in liver cancer.<br>Proposed aetiology: The aetiology of Signature 12 remains unknown.<br>Additional mutational features: Signature 12 exhibits a strong transcriptional strand-bias for T>C substitutions.
Signature W4	0.986239		
Signature W5	0.979987	Signature 19	Cancer types: Signature 19 has been found only in pilocytic astrocytoma.<br>Proposed aetiology: The aetiology of Signature 19 remains unknown.
Signature W6	0.955713		
Signature W7	0.941089		
Signature W8	0.925768	Signature 5	(See above)

Signature in transcribing regions of five samples shown in **Supplementary Figure 7a**	Stability	Most similar COSMIC signature[*]	Description of COSMIC signature[**]
Signature AS	0.91969	Signature 1	(See above)
Signature BS	0.945886	Signature 16	(See above)
Signature CS	0.904091	Signature 5	(See above)

Signature in different methylation level of five samples shown in **Supplementary Figure 9a**	Stability	Most similar COSMIC signature[*]	Description of COSMIC signature[**]
Signature X1	0.77187		
Signature X2	0.994669	Signature 16	(See above)
Signature X3	0.995489	Signature 12	(See above)
Signature X4	0.729329		
Signature X5	0.773439		
Signature X6	0.70885		
Signature X7	0.346684		
Signature X8	0.156446		

Signature in transcribing regions of 266 samples shown in **Supplementary Figure 10a**	Stability	Most similar COSMIC signature[*]	Description of COSMIC signature[**]
Signature WS1	0.996305	Signature 1	(See above)
Signature WS2	0.996798	Signature 16	(See above)
Signature WS3	0.976112		
Signature WS4	0.859419		
Signature WS5	0.922547	Signature 19	(See above)
Signature WS6	0.882345	Signature 1	(See above)
Signature WS7	0.925259	Signature 8	Cancer types: Signature 8 has been found in breast cancer and medulloblastoma.<br>Proposed aetiology: The aetiology of Signature 8 remains unknown.<br>Additional mutational features: Signature 8 exhibits weak strand bias for C>A substitutions and is associated with double nucleotide substitutions, notably CC>AA.

Signature in transcribing regions in different methylation level of five samples shown in **Supplementary Figure 12a**	Stability	Most similar COSMIC signature[*]	Description of COSMIC signature[**]
Signature XS1	0.471036		
Signature XS2	0.994987		
Signature XS3	0.871618		
Signature XS4	0.552125		
Signature XS5	0.407402		
Signature XS6	0.188623		
Signature XS7	-0.12131		

* Shown only for which the stability of the signature was more than 0.8 and the cosign similarity to COSMIC signature was more than 0.8.
** Referred from COSMIC website (http://cancer.sanger.ac.uk/cosmic/signatures).

Supplementary Table 5:
Difference of the odds ratio among viral feature

All states:

Signature	Similar COSMIC signature	HBV	NBNC	p-value*
W1	Sig. 1	1.289992243	0.77519846	0
W2	Sig. 16	0.871968704	1.146830151	1.84E-75
W3	Sig. 12	0.859206162	1.163865023	2.68E-198
W4		0.825098063	1.211977152	2.29E-244
W5	Sig. 19	0.997274572	1.002732876	0.745857438
W6		1.417168233	0.705632526	1.02E-304
W7		1.245542287	0.802863146	2.90E-277
W8	Sig. 5	0.898973315	1.112380071	3.09E-96

Signature	Similar COSMIC signature	HCV	NBNC	p-value*
W1	Sig. 1	1.058437696	0.944788724	8.10E-31
W2	Sig. 16	0.921316969	1.08540278	3.78E-38
W3	Sig. 12	0.843592593	1.18540633	0
W4		0.937048525	1.067180593	7.10E-41
W5	Sig. 19	1.101122083	0.908164513	1.66E-41
W6		1.069530079	0.934990067	4.78E-15
W7		1.163877472	0.859196972	4.11E-173
W8	Sig. 5	1.061564057	0.942006272	1.44E-43

Signature	Similar COSMIC signature	HCV	HBV	p-value*
W1	Sig. 1	0.820520669	1.218738343	0
W2	Sig. 16	1.056606785	0.946425874	2.25E-21
W3	Sig. 12	0.981790742	1.018546985	3.43E-06
W4		1.135712373	0.880504628	1.09E-178
W5	Sig. 19	1.104129407	0.905690939	1.18E-55
W6		0.754691762	1.325044277	0
W7		0.934440152	1.070159494	3.54E-50
W8	Sig. 5	1.180818811	0.846869978	0

Difference of odds ratio among viral feature in Active states:

Signature	Similar COSMIC signature	HBV	NBNC	p-value*
W1	Sig. 1	1.357791623	0.736490035	1.50E-76
W2	Sig. 16	0.767448726	1.303018646	2.49E-97
W3	Sig. 12	0.834966284	1.197653149	1.11E-30
W4		0.973459159	1.027264463	0.172307774
W5	Sig. 19	1.080292043	0.925675614	0.000305246
W6		1.48485724	0.673465417	9.78E-107
W7		1.322585684	0.75609468	1.35E-32
W8	Sig. 5	0.829851133	1.205035409	3.94E-26

Signature	Similar COSMIC signature	HCV	NBNC	p-value*
W1	Sig. 1	1.138202239	0.878578486	2.26E-18
W2	Sig. 16	0.808057574	1.237535581	7.53E-86
W3	Sig. 12	0.828130831	1.207538667	3.47E-44
W4		1.048315616	0.953911193	0.005018176
W5	Sig. 19	1.087421821	0.91960634	6.03E-06
W6		1.156843018	0.86442152	7.05E-19
W7		1.203537141	0.830884204	7.93E-19
W8	Sig. 5	1.181514284	0.846371486	6.00E-31

Signature	Similar COSMIC signature	HCV	HBV	p-value*
W1	Sig. 1	0.838293205	1.192900042	3.75E-48
W2	Sig. 16	1.052903702	0.949754472	1.62E-07
W3	Sig. 12	0.991848824	1.008218164	0.503645878
W4		1.076915858	0.928577653	4.97E-07
W5	Sig. 19	1.006618834	0.993424687	0.680898864
W6		0.779070804	1.283580382	3.25E-81
W7		0.909994589	1.098907633	2.73E-08
W8	Sig. 5	1.423788765	0.702351377	9.04E-161

*Using Fisher’s exact test

SupplementaryTable 6 | Epigenomic segment and methylation level of HBV integration site

Samples	HBV integration site		Epigenomic segment*			Gene Annotated**	Detected***
	chr	position	Segment	Methylation level (%)			
				Non-cancerous	Tumor		
HX14T	chr5	1,293,404<br>1,293,415	E14	88.1	54.9	TERT	SR<br>SR
HX25T	chr1	80,892,940<br>80,892,952<br>80,893,140	E15	74.4	63.2	-	SR<br>SR<br>PE
	chr5	1,298,624	E14	78.9	11.1	TERT	SR
	chr5	1,553,007	E15	84.3	49.3	-	SR
	chr5	16,264,243	E15	69.8	14.4	-	SR
	chr5	21,359,824<br>21,359,723	E15	73.3	14.2	-	SR<br>PE
	chr5	36,749,280	E14	79.0	36.0	-	SR
	chr5	62,473,245	E15	81.6	69.2	-	SR
	chr5	62,576,192<br>62,576,338	E15	71.5	59.1	-	SR<br>PE
	chr6	26,906,678	E15	90.3	81.3	GUSBP2	PE
HX28T	chr4	19,502,306	E15	80.0	62.0	-	SR
	chr4	42,950,249	E14	86.1	33.2	GRXCR1	SR
	chr7	61,083,517	E8	59.0	45.5	-	PE
	chr19	27,887,536	E9	56.4	42.9	-	PE
HX33T	chr5	1,296,402<br>1,296,477	E14	-	49.7	TERT	SR<br>SR
	chr10	42,391,526	E8	-	15.8	-	PE
	chr10	42,597,066	E8	-	14.4	-	PE
HX35T	chr3	162,048,403<br>162,048,599	E15	-	72.2	-	SR<br>PE
	chr5	1,295,188<br>1,295,224	E7	-	35.1	TERT	SR<br>SR
	chr10	112,718,180	E5	-	88.6	SHOC2	SR
	chr13	82,130,229<br>82,130,447	E15	-	63.1	-	SR<br>PE

*The 15 epigenomic segments of adult liver cell defined in ENCODE project (see Supplementary Fig. 2 and ref 7)
** Including 20k bases from gene transcribing regions
*** PE: center position of cluster inferred with paired end reads; SR: determined with single read

Supplementary Table 7
GSEA Gene Sets investigation using C5*

Overlap Results	
Collection(s):	C5
# overlaps shown:	10
# genesets in collections:	1,454
# genes in comparison (n):	209
# genes in universe (N):	45,956

Gene Set Name	# Genes in Gene Set (K)**	Description	# Genes in Overlap (k)**	k/K**	p-value	FDR q-value
CYTOPLASM	2,131	Genes annotated by the GO term GO:0005737. All of the contents of a cell excluding the plasma membrane and nucleus, but including other subcellular structures.	35	0.0164	4.56E-11	6.62E-08
CYTOPLASMIC_PART	1,383	Genes annotated by the GO term GO:0044444. Any constituent part of the cytoplasm, all of the contents of a cell excluding the plasma membrane and nucleus, but including other subcellular structures.	27	0.0195	2.39E-10	1.73E-07
NUCLEOBASENUCLEOSIDENUCLEOTIDE_AND_NUCLEIC_ACID_METABOLIC_PROCESS	1,244	Genes annotated by the GO term GO:0006139. The chemical reactions and pathways involving nucleobases, nucleosides, nucleotides and nucleic acids.	24	0.0193	3.23E-09	1.57E-06
NUCLEUS	1,430	Genes annotated by the GO term GO:0005634. A membrane-bounded organelle of eukaryotic cells in which chromosomes are housed and replicated. In most cells, the nucleus contains all of the cell's chromosomes except the organellar chromosomes, and is the site of RNA synthesis and processing. In some species, or in specialized cell types, RNA metabolism or DNA replication may be absent.	25	0.0175	1.05E-08	3.81E-06
BIOPOLYMER_METABOLIC_PROCESS	1,684	Genes annotated by the GO term GO:0043283. The chemical reactions and pathways involving biopolymers, long, repeating chains of monomers found in nature e.g. polysaccharides and proteins.	27	0.016	1.57E-08	3.84E-06
MACROMOLECULAR_COMPLEX	945	Genes annotated by the GO term GO:0032991. A stable assembly of two or more macromolecules, i.e. proteins, nucleic acids, carbohydrates or lipids, in which the constituent parts function together.	20	0.0212	1.58E-08	3.84E-06
PROTEIN_COMPLEX	816	Genes annotated by the GO term GO:0043234. Any protein group composed of two or more subunits, which may or may not be identical. Protein complexes may have other associated non-protein prosthetic groups, such as nucleic acids, metal ions or carbohydrate groups.	18	0.0221	4.62E-08	9.59E-06
PROTEIN_METABOLIC_PROCESS	1,231	Genes annotated by the GO term GO:0019538. The chemical reactions and pathways involving a specific protein, rather than of proteins in general. Includes protein modification.	22	0.0179	5.83E-08	1.06E-05
CELL_DEVELOPMENT	577	Genes annotated by the GO term GO:0048468. The process whose specific outcome is the progression of the cell over time, from its formation to the mature structure. Cell development does not include the steps involved in committing a cell to a specific fate.	15	0.026	7.89E-08	1.27E-05
CELLULAR_PROTEIN_METABOLIC_PROCESS	1,117	Genes annotated by the GO term GO:0044267. The chemical reactions and pathways involving a specific protein, rather than of proteins in general, occurring at the level of an individual cell. Includes protein modification.	20	0.0179	2.34E-07	3.40E-05

* C5 is GO gene sets consist of genes annotated by the same GO terms (http://software.broadinstitute.org/gsea/msigdb/index.jsp) .
** K is the number of genes in the set from C5. k is the number of genes in the intersection of the query set with a set from C5.

Supplementary Table 8
GSEA Gene Sets investigation using BIOCARTA, KEGG and REACTOME in C2*

Overlap Results	
Collection(s):	CP:BIOCARTA, CP:KEGG, CP:REACTOME
# overlaps shown:	8
# genesets in collections:	1,077
# genes in comparison (n):	209
# genes in universe (N):	45,956

Gene Set Name	# Genes in Gene Set (K)**	Description	# Genes in Overlap (k)**	k/K**	p-value	FDR q-value
REACTOME_RECYCLING_OF_BILE_ACIDS_AND_SALTS	11	Genes involved in Recycling of bile acids and salts	4	0.3636	1.34E-07	1.44E-04
REACTOME_BILE_ACID_AND_BILE_SALT_METABOLISM	27	Genes involved in Bile acid and bile salt metabolism	4	0.1481	6.72E-06	3.62E-03
REACTOME_TRANSPORT_OF_VITAMINS_NUCLEOSIDES_AND_RELATED_MOLECULES	31	Genes involved in Transport of vitamins, nucleosides, and related molecules	4	0.129	1.19E-05	4.01E-03
REACTOME_TRANSPORT_OF_ORGANIC_ANIONS	11	Genes involved in Transport of organic anions	3	0.2727	1.49E-05	4.01E-03
REACTOME_BIOLOGICAL_OXIDATIONS	139	Genes involved in Biological oxidations	6	0.0432	4.47E-05	9.63E-03
REACTOME_TRANSMEMBRANE_TRANSPORT_OF_SMALL_MOLECULES	413	Genes involved in Transmembrane transport of small molecules	9	0.0218	1.27E-04	2.29E-02
KEGG_METABOLISM_OF_XENOBIOTICS_BY_CYTOCHROME_P450	70	Metabolism of xenobiotics by cytochrome P450	4	0.0571	3.01E-04	4.52E-02
KEGG_DRUG_METABOLISM_CYTOCHROME_P450	72	Drug metabolism - cytochrome P450	4	0.0556	3.36E-04	4.52E-02

* C2 is curated gene sets from online pathway databases, publications in PubMed, and knowledge of domain experts (http://software.broadinstitute.org/gsea/msigdb/index.jsp) .
** K is the number of genes in the set from BIOCARTA in C2, KEGG and REACTOME. k is the number of genes in the intersection of the query set with a set from BIOCARTA, KEGG and REACTOME in C2.

Supplementary Table 9:
Comparison of frequency of rearrangements in human and HBV genome

	Human genome (reference size: 3,095,677,412)					HBV genome (reference size: 3,215)				
	HX14T	HX25T	HX28T	HX33T	HX35T	HX14T	HX25T	HX28T	HX33T	HX35T
Number of rearrangements	54	60	100	41	81	1	14	9	2	1
Base number of covered region by whole-genome sequencing	2,858,055,467	2,860,307,592	2,860,033,430	2,857,167,753	2,856,220,312	1,865	3,143	3,213	3,063	3,214
Depth	42.4	62.4	53.5	24.9	25.2	25.0	268.1	93.1	20.0	19.7
Ratio of depth vs. human genome	-	-	-	-	-	0.59	4.29	1.74	0.81	0.78
Number of rearrangement adjusted to depth of human genome*	-	-	-	-	-	1.69	3.26	5.17	2.48	1.28
Frequency (Number of rearrangement /Mb)	0.0189	0.0210	0.0350	0.0143	0.0284	907.8**	1037.6**	1609.4**	811.0**	397.6**
Ratio of frequency vs. human genome	-	-	-	-	-	48,046	49,462	46,030	56,516	14,022

*It was considered that multiple copies of HBV genome integrated into human genome.
***P* < 2.2e-16 using chi-squared test, compared with the frequency of human genome.

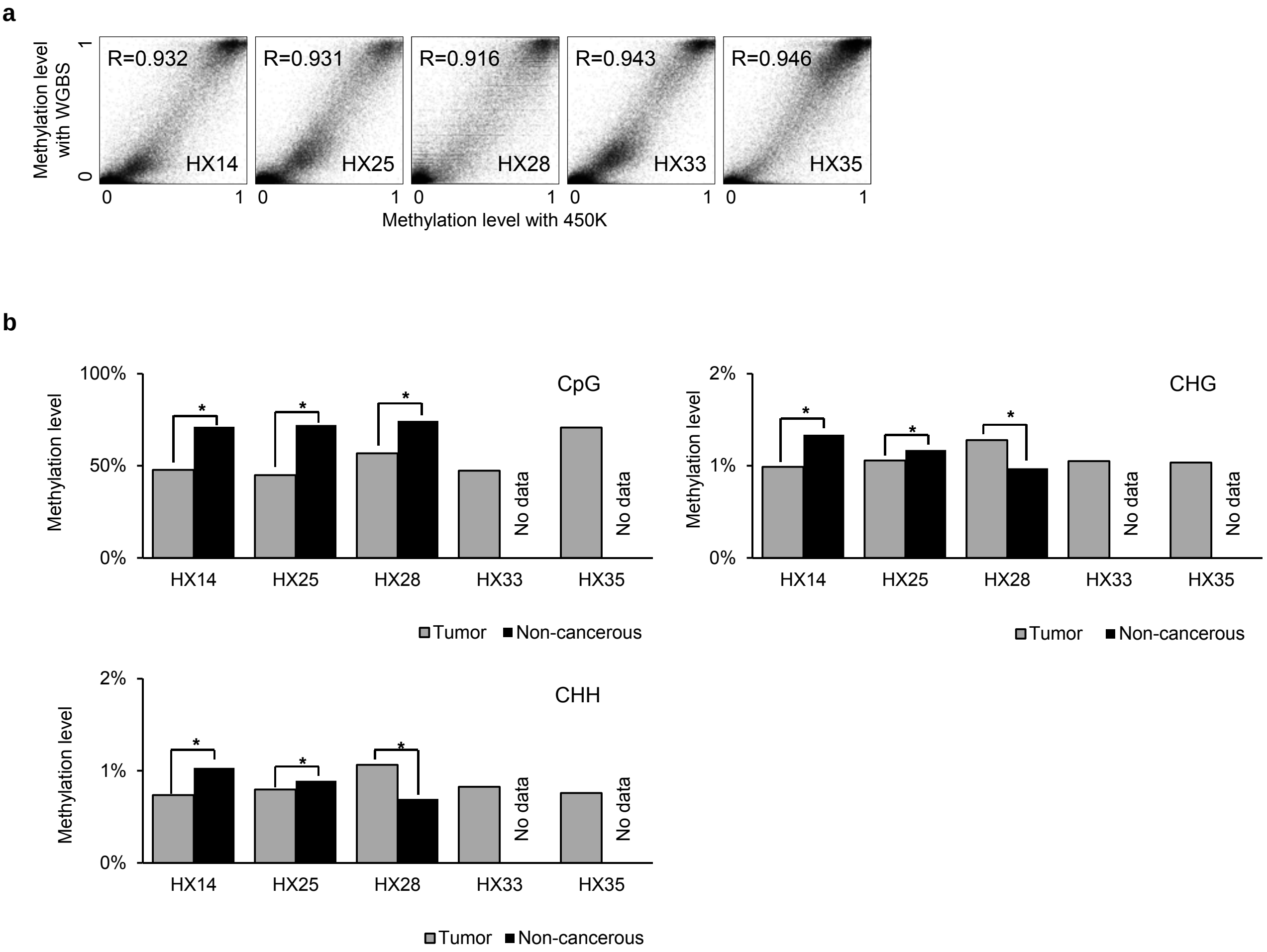

Supplementary Figure 1 | Detection of methylated cytosines by whole-genome bisulfite sequencing (WGBS).
a, Comparison of the methylation levels of tumor samples between WGBS and Infinium HumanMethylation450K. The correlations were 0.91 – 0.95 (*P* < 2.2e-16 with Pearson's correlation test). The x-axis indicates the beta-value of HumanMethylation450; the y-axis indicates the methylation level of WGBS. **b**, Comparison of cytosine methylation levels in the CpG, CHG (where H = A, T or C), or CHH site supported at least five reads between tumor and non-cancerous samples. The methylation level of the CpG site in tumor tissue was significantly lower (**P* < 2.2e-16 using Fisher's exact t-test) than that in non-cancerous tissue.

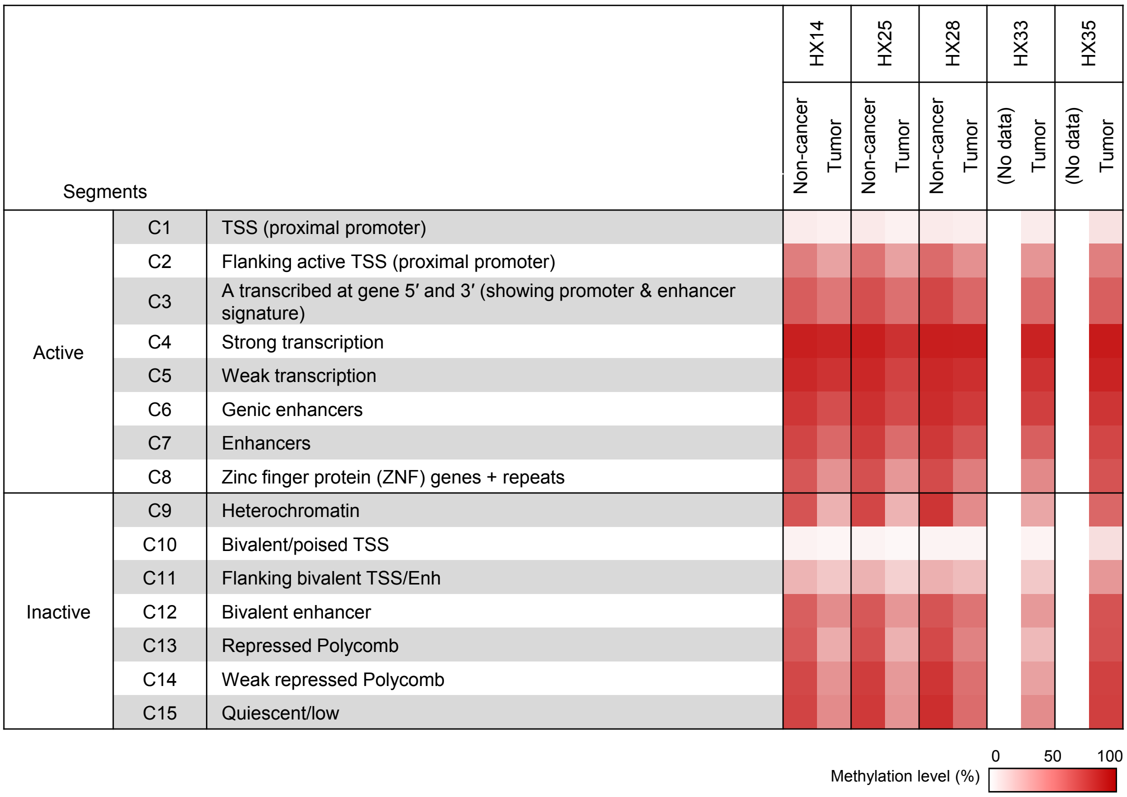

Supplementary Figure 2 | The 15 epigenomic segments of the human genome defined in ENCODE[7] and the methylation levels in five hepatocellular carcinoma (HCC) samples.
ENCODE shows the data of the HepG2 genome regions defined according to the 15 epigenomic segments (http://egg2.wustl.edu/roadmap/web_portal/). Whole-genome bisulfite sequencing (WGBS) of five HCC samples was used to calculate the methylation levels of the segments in each samples. WGBS was not performed in the non-cancer samples of HX33 and HX35.

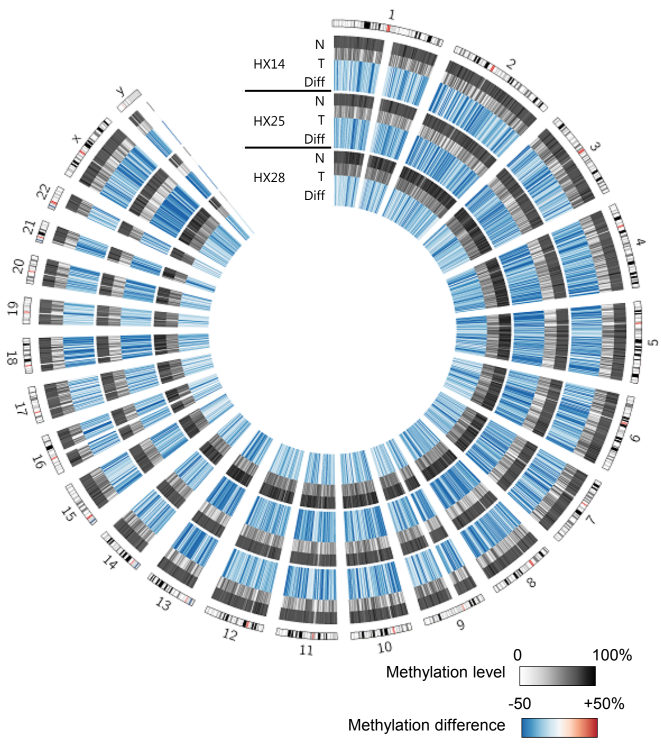

Supplementary Figure 3 | A global view of the methylation levels of tumor and non-cancerous genomes. The methylation levels of three paired samples were calculated for each million bases using whole-genome bisulfite sequencing. N: non-cancerous; T: tumor; Diff: the difference of the methylation level between non-cancerous and tumor paired samples. Most of the genomes of the three tumors were hypo-methylated (blue), and a few hyper-methylated regions (red) were observed.

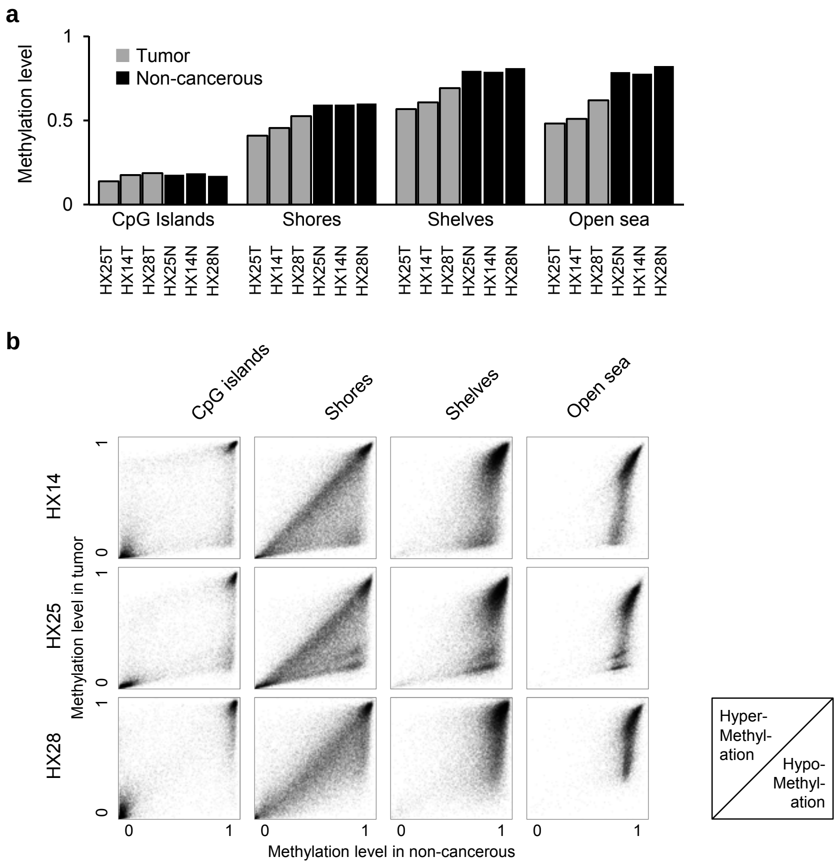

Supplementary Figure 4 | Hypo-methylation in CpG shores (regions 0-2 kb from CpG islands), CpG shelves (2–4 kb from the islands), and open seas.

a, Average methylation levels of CpG islands, shores, shelves, or open seas of three paired samples. **b**, Difference in the methylation levels between non-cancerous and tumor genomes. The x-axis indicates the methylation level of non-cancerous tissues; the y-axis indicates the methylation level of tumor tissues.

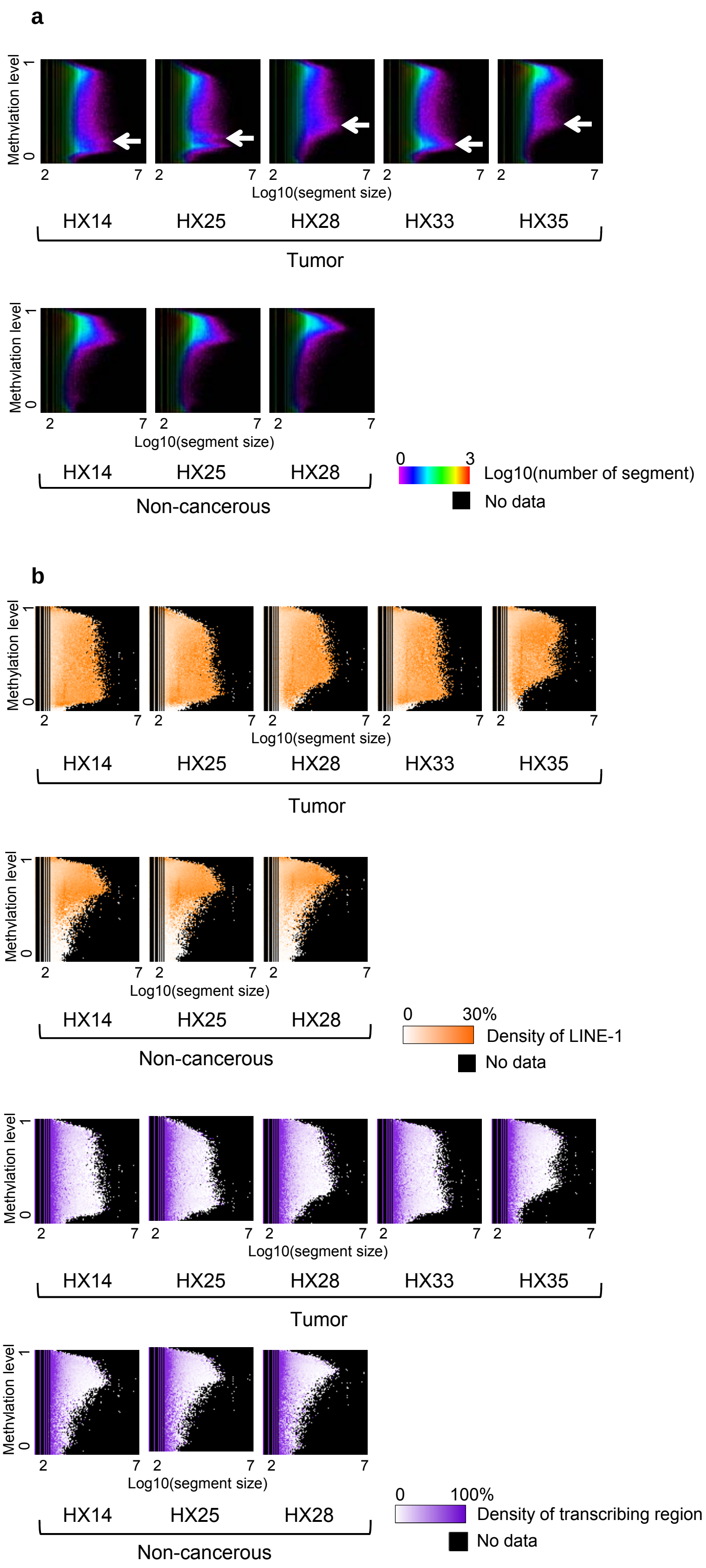

Supplementary Figure 5 | Methylation levels and sizes of the genomic regions of tumor and non-cancerous samples according to the definition of the 15 epigenomic segments.

a. Five tumor and three non-cancerous liver genomes were delimited to approximately 561,000 regions according to the 15 epigenomic segments of HepG2 which defined by ENCODE project[7]. The regions were plotted according to methylation level (y-axis) and size (x-axis). The arrow indicates the fraction of tumor-specific hypo-methylated regions. **b**. The density of LINE-1 or transcribing regions. Orange indicates the density of LINE-1 and purple indicates the density of transcribed regions per million bases calculated for each fraction of the images in a, which were divided into 100 × 100 areas.

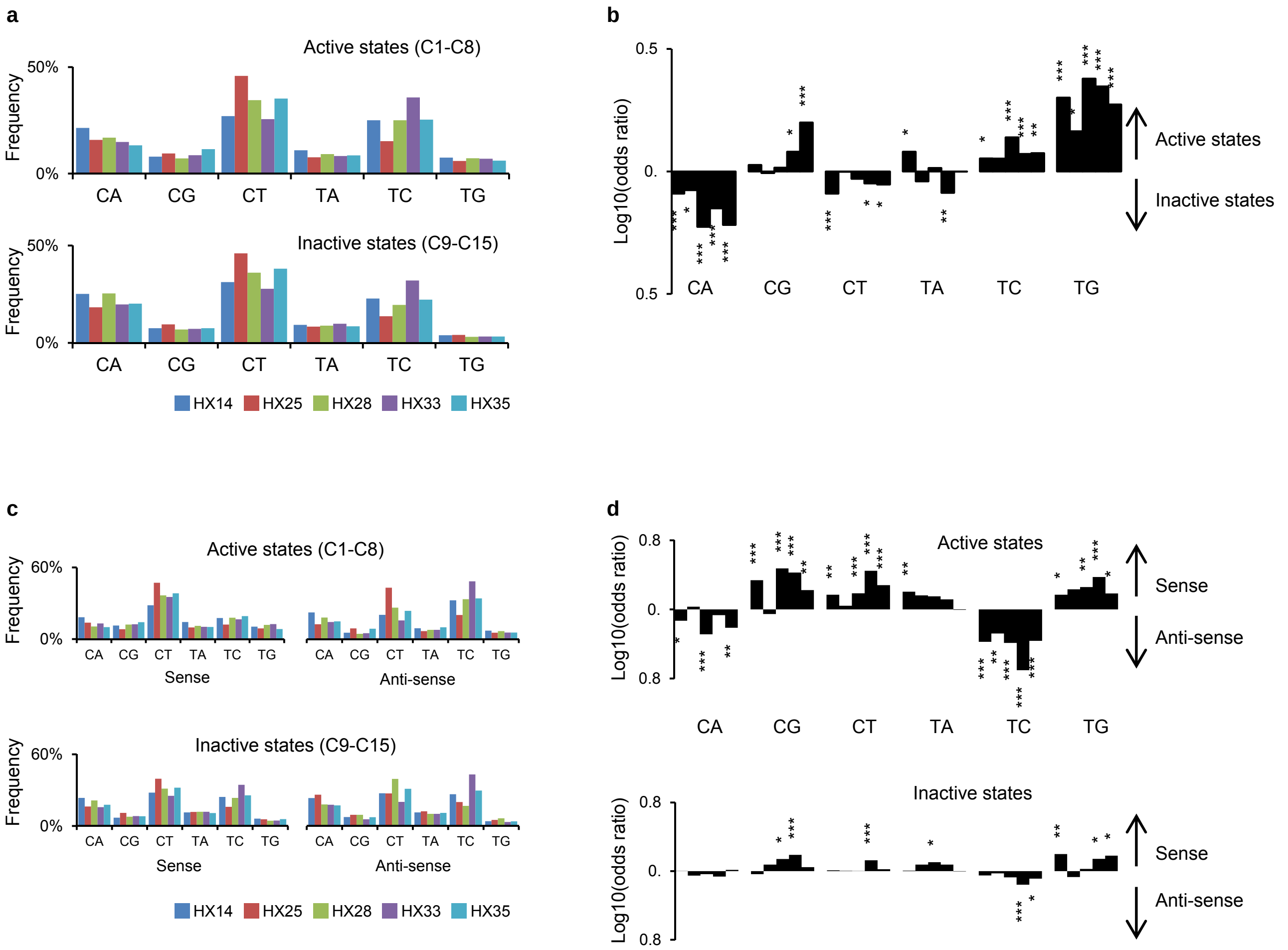

Supplementary Figure 6 | Six-substitution pattern of five hepatocellular carcinoma samples in active or inactive chromatin states.

a, The frequencies of six substitution patterns within active or inactive chromatin areas in five tumor genomes. **b**, Odds ratio of the frequencies of six substitution patterns within active vs. inactive areas. *$P < 0.05$, **$P < 0.005$, ***$P < 0.0005$ calculated using Fisher's exact t-test. **c,** The frequencies of six-substitution pattern in the sense or anti-sense strand within active or inactive chromatin areas. **d**, The odds ratio of the frequencies of six substitution patterns in sense vs. in anti-sense strand. *$P < 0.05$, **$P < 0.005$, ***$P < 0.0005$ calculated using Fisher's exact t-test.

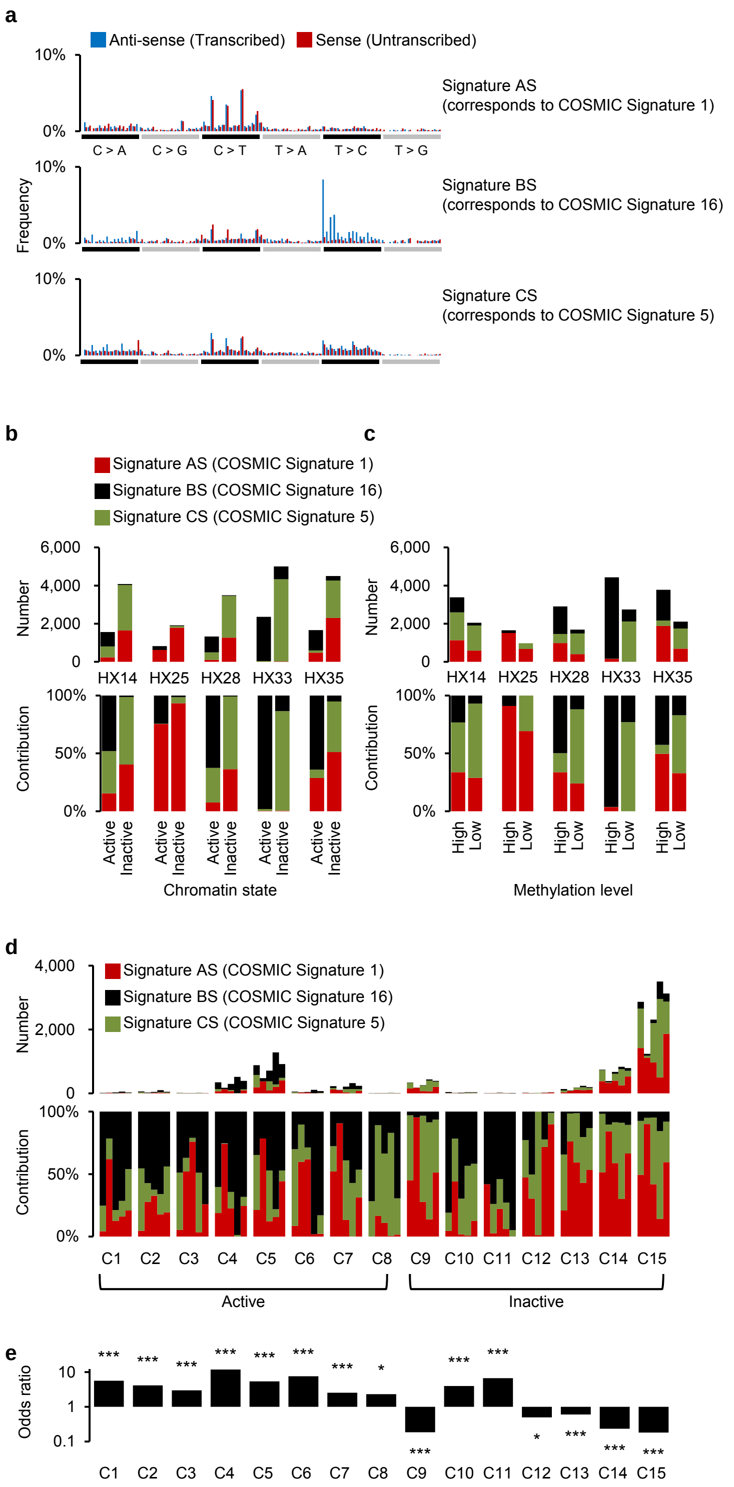

Supplementary Figure 7 | Signature analysis of transcribed regions in different chromatin states or different methylation levels

Three mutational signatures of 192 substitution patterns considering the transcribing strand in transcribed regions. The substitutions in transcribed regions were selected from the substitution list analyzed in **Fig. 3a-e**. **a**. The frequencies of three mutational signatures in transcribed and untranscribed regions within active or inactive chromatin areas. The stability of the signature and the most similar COSMIC Signature are shown in **Supplementary Table 4**. In signature BS2, associated with COSMIC Signature 16, the frequency of the sense side (shown by red) was observed slightly. **b**. Comparison of the numbers and the contributions of mutational signatures within the active and inactive chromatin areas. **c**. Comparison of the numbers and the contributions of mutational signatures within highly methylated and low methylated genomes. **d**. The numbers and the contributions of mutational signatures in 15 epigenomic segments. **e**. Odds ratio of Signature BS, associated to COSMIC Signature 16, in a particular epigenomic segment compared with others. **P* < 0.05, ***P* < 0.005, ****P* < 0.0005 tested with Fisher's t-test.

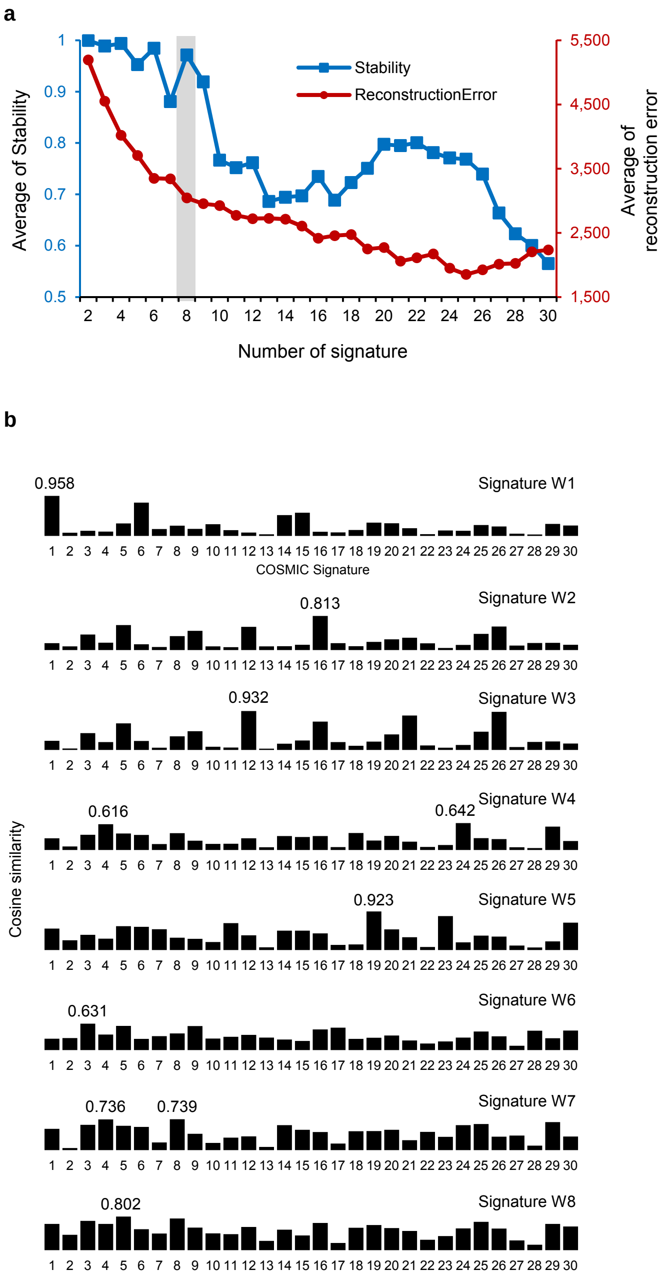

Supplementary Figure 8 | Stability and similarity of mutational signatures of 266 hepatocellular carcinomas.
a, Average of the stability and reconstruction error of mutational signature analysis. The numbers of signatures from 2 to 30 were tested, and the eight indicated in gray were used for the analysis in **Figure 4a-c**. **b**, Cosine similarities of the eight signatures to the 30 COSMIC signatures.

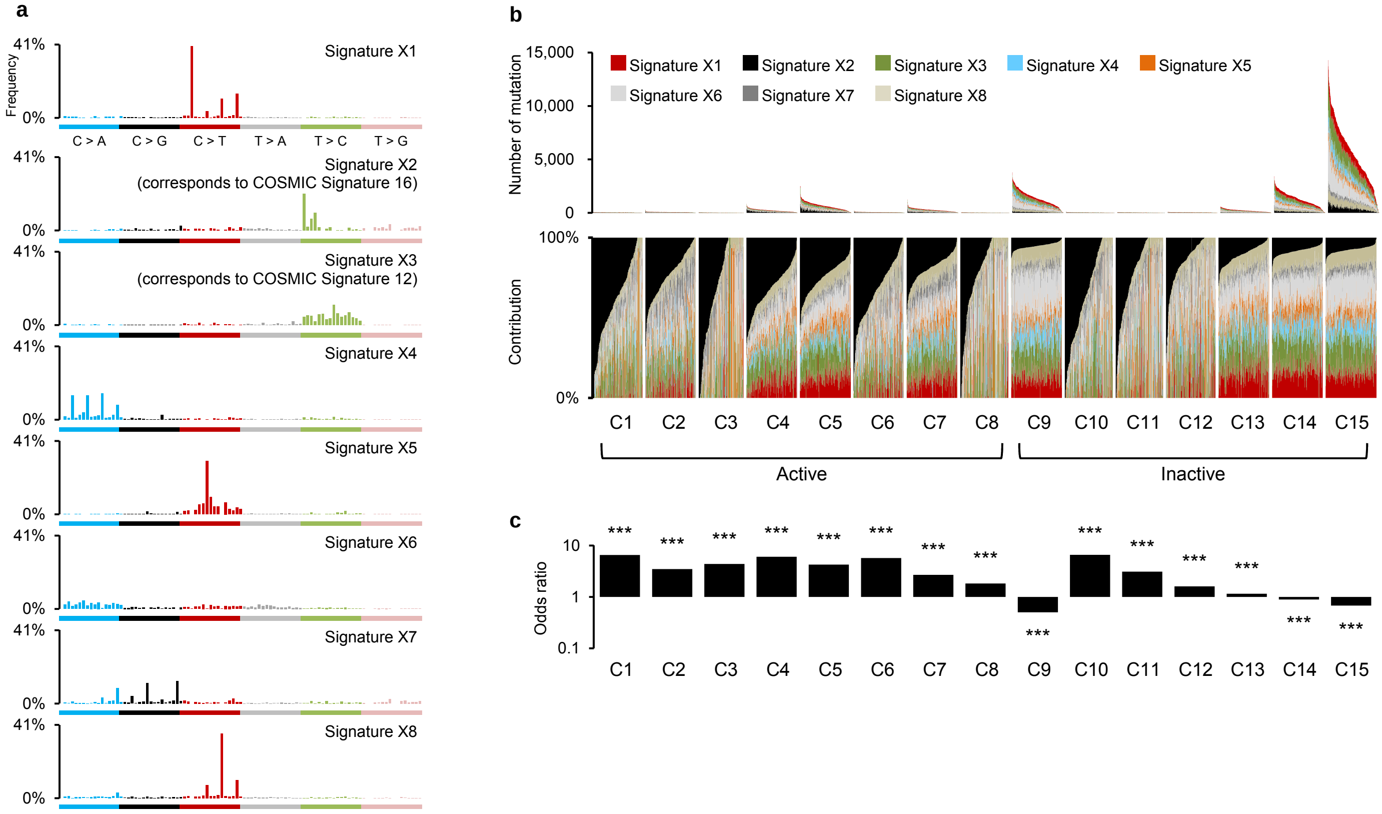

Supplementary Figure 9 | Mutational signature analysis using 15 epigenomic segments of 266 hepatocellular carcinomas (HCCs).

In **Fig. 4**, the contributions of the mutational signatures were compared between active and inactive chromatin areas. To investigate in detail, here, the same mutational data were compared according to the 15 epigenomic segments. **a**, Signatures identified from the 15 epigenomic segments of 266 HCCs. These signatures were similar to those in Fig. 4a; however the stabilities of some signatures were low because the number of mutations per segment was small in some samples (**Supplementary Table 4**). **b**, The contribution of the signatures shown in a. **c**, Odds ratio of Signature X2, associated with COSMIC Signature 16, in a particular segment compared with the others. **P* < 0.05, ***P* < 0.005, ****P* < 0.0005 using Fisher's exact t-test.

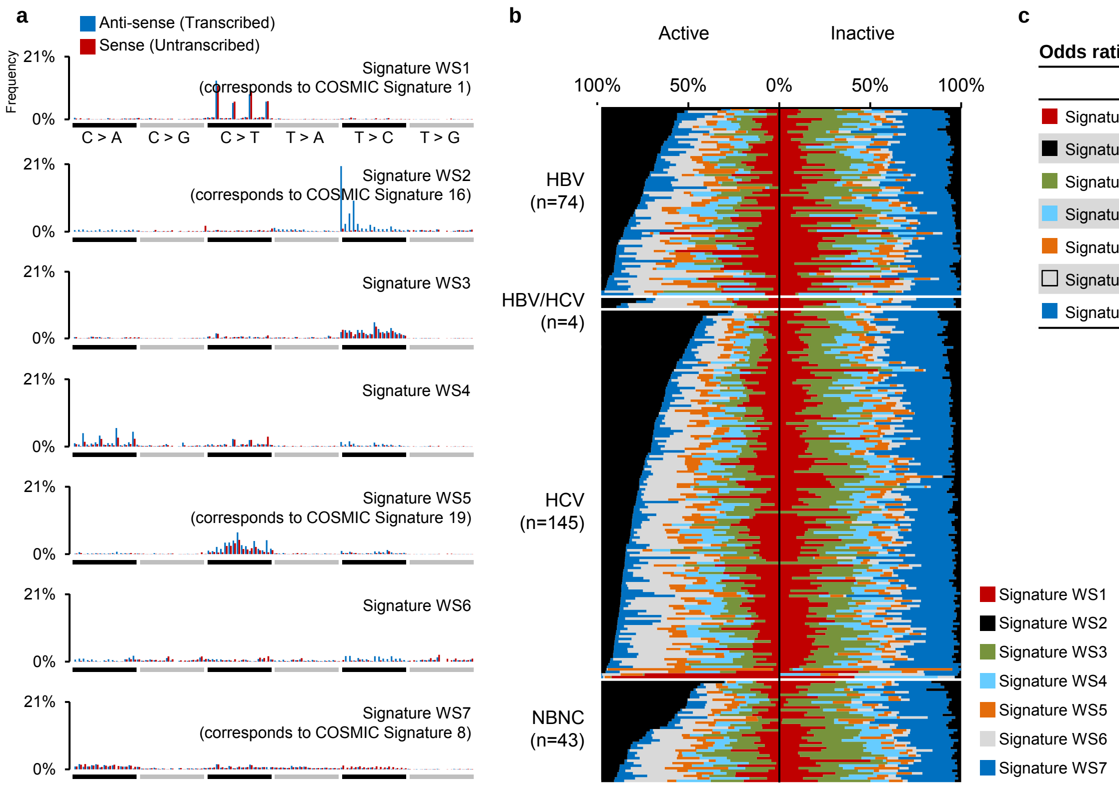

c

Odds ratio of signature

	Active	Inactive
Signature WS1	0.778525	1.28448
Signature WS2	7.184963	0.13918
Signature WS3	0.493214	2.027517
Signature WS4	0.782019	1.278741
Signature W55	1.208244	0.827648
Signature WS6	3.609995	0.277009
Signature WS7	0.188128	5.315524

p-value < 2.2e-16, Fisher's exact test

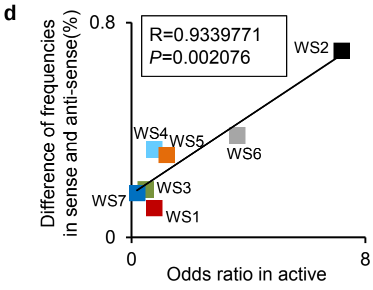

Supplementary Figure 10 | Mutational signatures of 266 hepatocellular carcinomas (HCCs) in transcribed regions. Seven mutational signatures of 192 substitution patterns of 266 HCCs according to the transcribing strand in transcribed regions. The substitutions in transcribed regions were selected from the substitution list in **Fig. 4a-c**. **a**. Frequencies of mutational signatures in transcribed and untranscribed regions within active or inactive chromatin areas. The stability of the signatures and the similarities to the COSMIC signature are shown in **Supplementary Table 4**. Low scale data and the odds ratio of frequencies in each strand are shown in **Supplementary Fig. 11**. **b**. The contributions of mutational signatures in active and inactive chromatin areas. **c**. Odds ratio of the contribution of each mutational signature in active or inactive chromatin areas. **d**. Correlation between the intensities of strand bias and the contribution in active chromatin areas of each mutational signature. *P* = 0.002076 Pearson's correlation test.

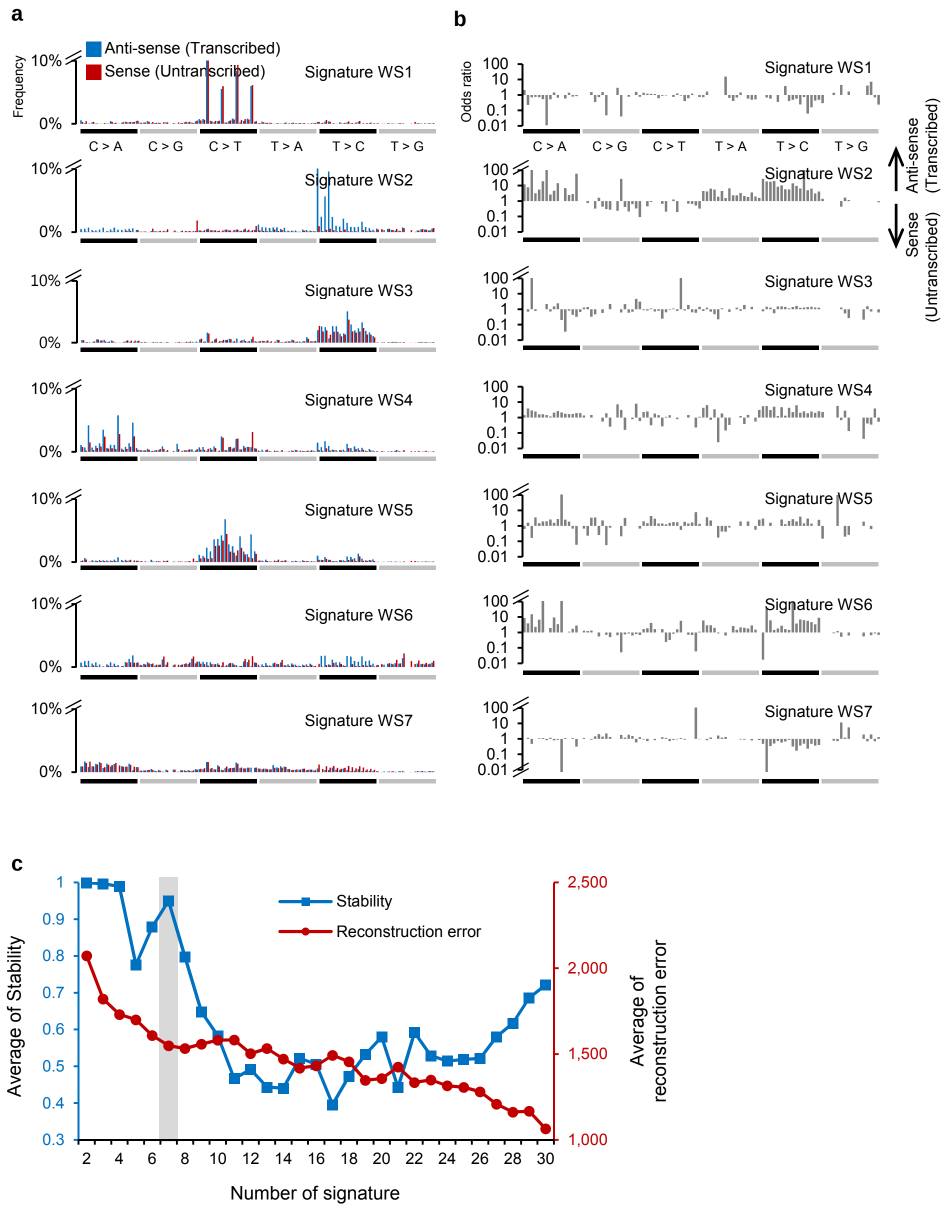

Supplementary Figure 11 | Mutational signatures of 266 hepatocellular carcinomas in transcribing regions. **a**, Expanded low scale data of **Supplementary Fig. 10a**. In signature WS2, associated with COSMIC Signature 16, the frequency of the sense side (shown by red) was observed slightly. **b**, The odds ratio of the frequencies in the anti-sense vs. sense strands. The odds ratio with $P \geq 0.05$ is not shown. **c**, Average stability and reconstruction error of the signatures shown in **Supplementary Fig. 10a-c.** The number of signatures used for the analysis in **Supplementary Fig. 10** is indicated in gray.

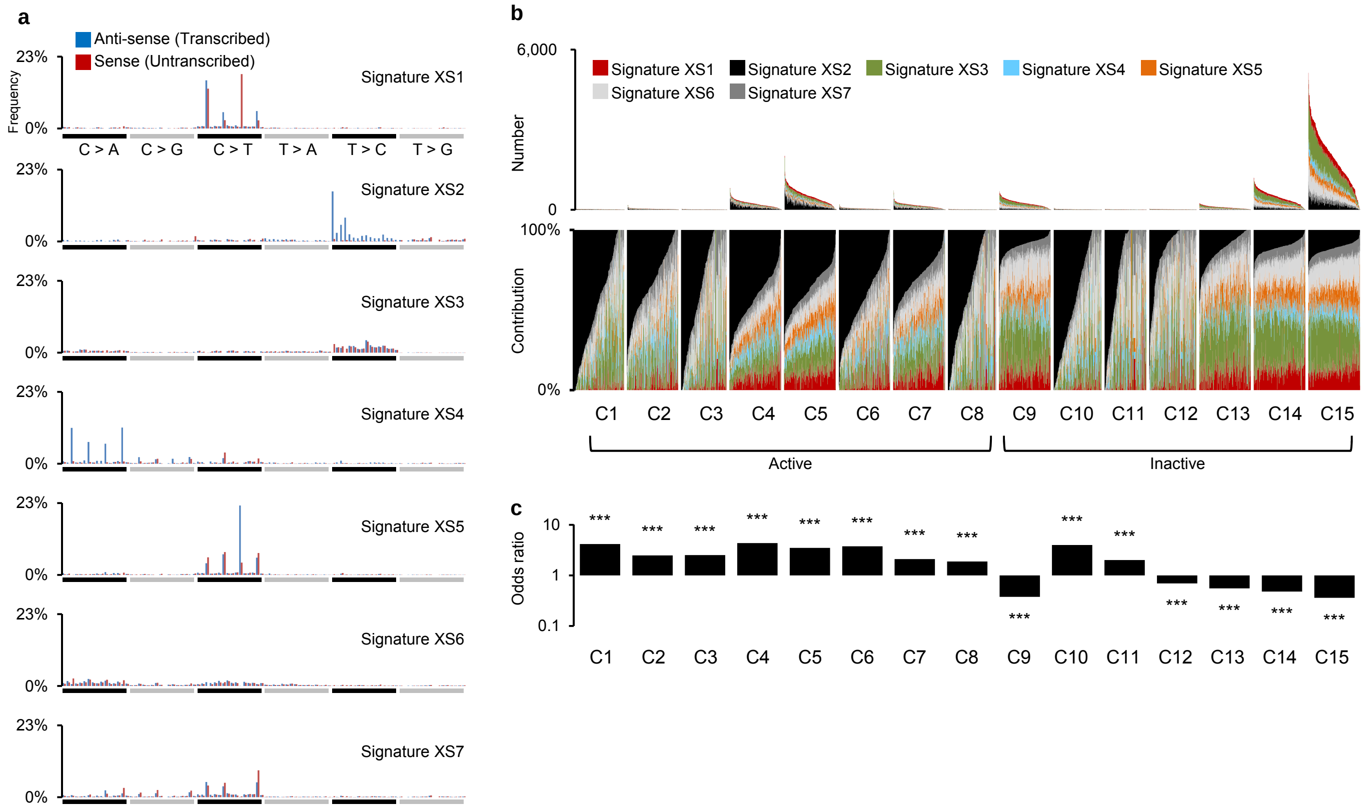

Supplementary Figure 12 | Mutational signature analysis using the 15 epigenomic segments of 266 hepatocellular carcinomas (HCCs) in transcribed regions.

In **Supplementary Fig. 10**, mutational signatures were compared between active and inactive chromatin areas. To investigate in detail, here, the same mutation data were compared with the 15 epigenomic segments. **a**, Signatures identified from 15 epigenomic segments of 266 HCCs. The stabilities of some signatures were low because the number of mutations per segment was small in some samples (**Supplementary Table 4**). **b**, The contribution of the signatures shown in a. **c**, Odds ratio of Signature XS2 in a particular segment compared with the others. *P <0.05, ** P <0.005, *** P <0.0005 using Fisher's exact test.

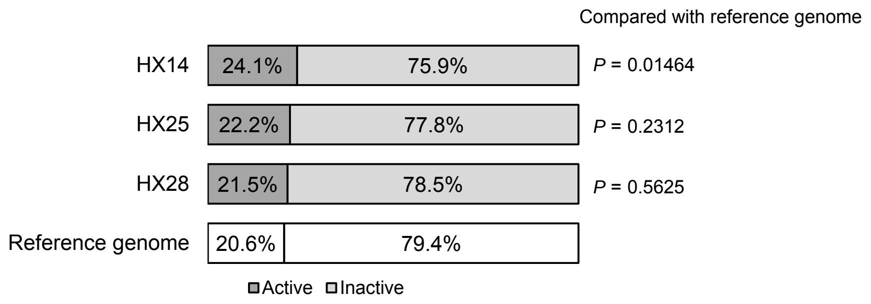

Supplementary Figure 13 | Structural alterations of LINE-1 in human genomes.
Rearrangement of LINE-1 in active or inactive chromatin areas in three HCCs. *P*-value was calculated with Chi-squared test.

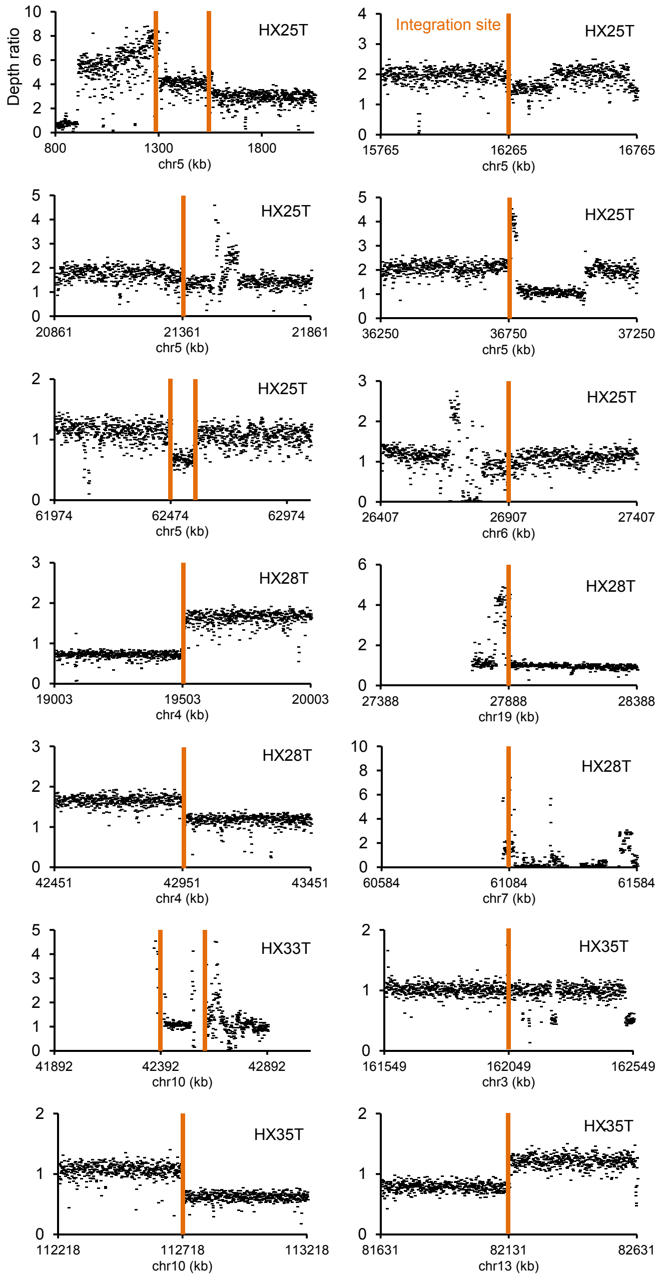

Supplementary Figure 14 | Copy number alterations at HBV integration sites in HCC samples.
The ratio of the sequence depth of tumor genomes to non-cancerous genomes in one million bases around HBV integration site. Copy number alterations were observed in 17 of 21 the HBV integration sites (**Supplementary Table 6**).

a

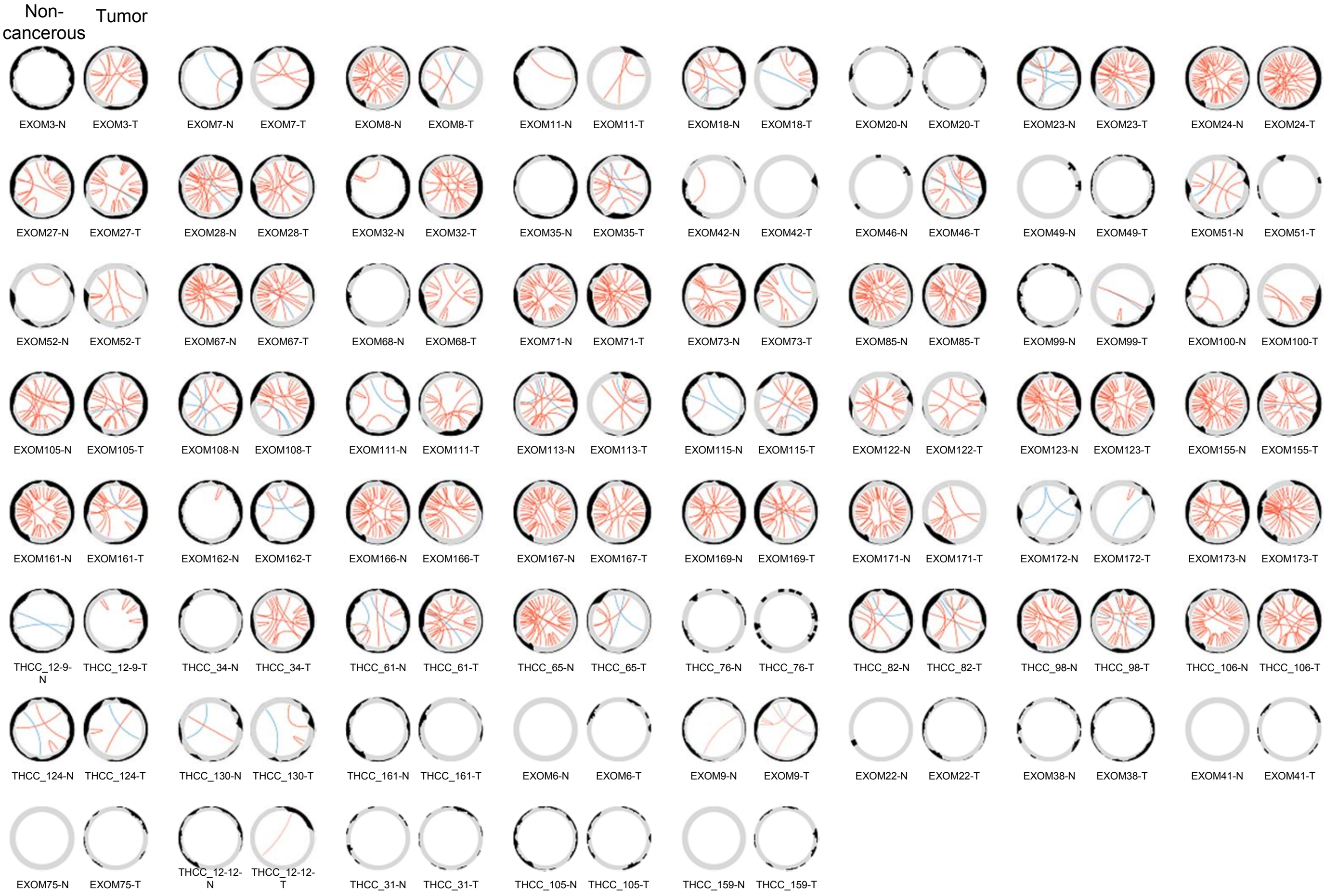

b

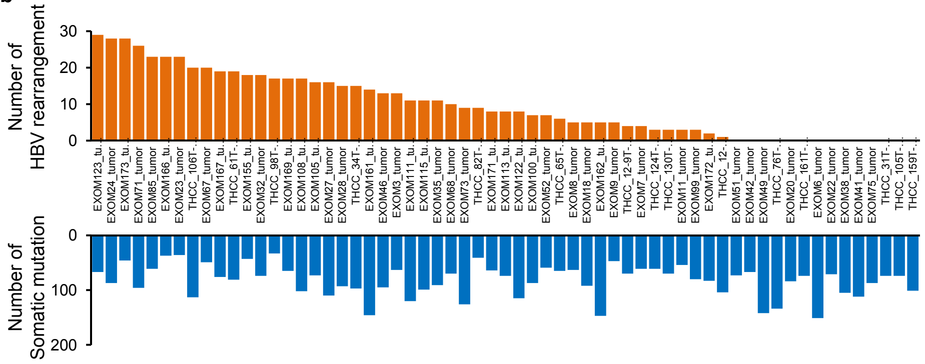

c

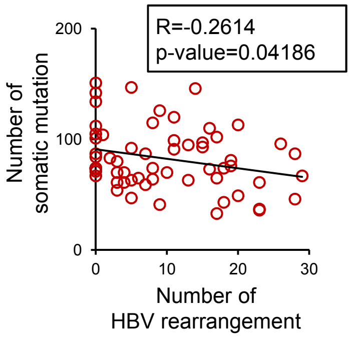

d

Gene mutated	# sample (total)		p-value*
	# HBV rearrangement		
	≥5	<5	
	(39)	(22)	
TERT	21	13	0.791
TP53	21	8	0.2857
CTNNB1	10	6	1
AXIN1	8	2	0.4772
ARID2	4	1	0.6451
CDKN2A	1	4	0.05238
ARID1A	2	2	0.6147
CCND1	3	1	1
FGF19	1	3	0.2904

*Compared with total number of sample, using Fisher's t test

Supplementary Figure 15 | HBV rearrangements in HCC samples. **a.** HBV genome rearrangements are represented by circular plots. The circle shows the HBV genome (3,215 bp). The red line shows improper direction paired-reads, and blue shows a longer distance than the fragment length. Copy number alterations are indicated in black. **b**. The distribution of the number of HBV rearrangements (upper panel) and the number of somatic mutations (lower panel) in 61 HCC samples. The samples are shown in descending order according to the number of HBV rearrangements. **c**. Correlation between HBV rearrangements and somatic mutations in the 61 HCC samples shown in b. *P*-value was calculated using Pearson's correlation test. **d**. Relationship between the presence of driver gene mutations and the number of rearrangements of the HBV genome. The 61 tumors were classified into groups with high (five or more rearrangements) and low (less than five rearrangements) rearrangement. No significant relationship with the presence of driver gene mutations was detected. *P*-value was calculated using Fisher's test.
