# Supplementary material for: Epigenetic landscape influences the liver cancer genome architecture
Source: Nat Commun. 2018 Apr 24;9:1643. doi: 10.1038/s41467-018-03999-y (PMC5915380; doi:10.1038/s41467-018-03999-y)
Supplement: Supplementary file 2 — Description of Additional Supplementary Files [file 41467_2018_3999_MOESM2_ESM.pdf]

Description of Additional Supplementary Files

Supplementary Data 1.

List of rearrangements in the five hepatocellular carcinoma samples analysed.
